# Thermoresponsive Block Copolymer Core–Shell Nanoparticles with Tunable Flow Behavior in Porous Media

**DOI:** 10.1021/acsami.2c15024

**Published:** 2022-11-19

**Authors:** Matthieu P. J. Miclotte, Spyridon Varlas, Carl D. Reynolds, Bilal Rashid, Emma Chapman, Rachel K. O’Reilly

**Affiliations:** † School of Chemistry, University of Birmingham, Edgbaston, Birmingham B15 2TT, U.K.; ‡ BP Exploration Operating Company Ltd., Sunbury-on-Thames, Middlesex TW16 7LN, U.K.

**Keywords:** thermoresponsive nanoparticles, polymerization-induced self-assembly, critical solution temperature, flow, sandpack, porous media

## Abstract

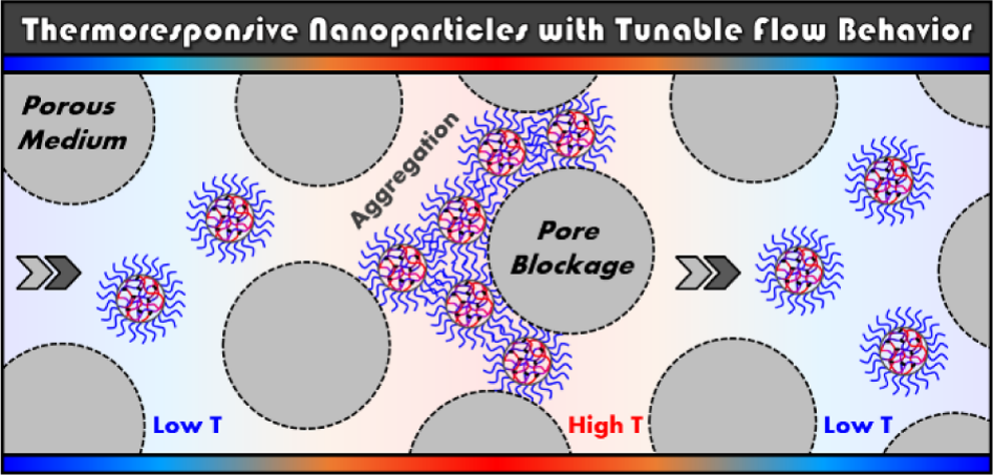

With the purpose of investigating new polymeric materials as potential flow modifiers for their future application in enhanced oil recovery (EOR), a series of amphiphilic poly(di(ethylene glycol) methyl ether methacrylate-*co*-oligo(ethylene glycol) methyl ether methacrylate) [P(DEGMA-*co*-OEGMA)]-based core–shell nanoparticles were prepared by aqueous reversible addition–fragmentation chain transfer-mediated polymerization-induced self-assembly. The developed nano-objects were shown to be thermoresponsive, demonstrating a reversible lower-critical solution temperature (LCST)-type phase transition with increasing solution temperature. Characterization of their thermoresponsive nature by variable-temperature UV–vis and dynamic light scattering analyses revealed that these particles reversibly aggregate when heated above their LCST and that the critical transition temperature could be accurately tuned by simply altering the molar ratio of core-forming monomers. Sandpack experiments were conducted to evaluate their pore-blocking performance at low flow rates in a porous medium heated at temperatures above their LCST. This analysis revealed that particles aggregated in the sandpack column and caused pore blockage with a significant reduction in the porous medium permeability. The developed aggregates and the increased pressure generated by the blockage were found to remain stable under the injection of brine and were observed to rapidly dissipate upon reducing the temperature below the LCST of each formulation. Further investigation by double-column sandpack analysis showed that the blockage was able to reform when re-heated and tracked the thermal front. Moreover, the rate of blockage formation was observed to be slower when the LCST of the injected particles was higher. Our investigation is expected to pave the way for the design of “smart” and versatile polymer technologies for EOR applications in future studies.

## Introduction

Stimuli-responsive, or “smart”, polymer materials have found a wide variety of applications over the past few decades, particularly due to the wide variety of external stimuli that can be used to induce the desired response, such as pH,^1^ light irradiation,^2^ or temperature,^3^ among others. Consequently, these materials have played an important role in the development of advanced technologies, whereby they have been successfully applied in drug delivery,^4,5^ protein purification,^6^ interactive coatings,^7,8^ and tissue engineering.^9,10^ Thermo-sensitive polymers are a common class of responsive materials that undergo a reversible change in their solubility at a specific temperature known as critical transition temperature. This behavior is typically used to classify thermoresponsive polymers into one of two categories depending on whether they phase-separate from the solvent matrix with increasing temperature, exhibiting a lower-critical solution temperature (LCST), or become solvent-miscible with increasing temperature, exhibiting an upper-critical solution temperature (UCST).^11,12^ To date, the vast majority of literature reports have focused on the utilization of thermoresponsive polymers presenting LCST-type transitions in aqueous solutions as UCST-type behavior is rather scarcely encountered owing to its high sensitivity to small variations in pH, ionic strength, and polymer composition.^13−17^

Among the existing plethora of LCST-type polymers, probably the most widely investigated in this field is poly(*N*-isopropylacrylamide) (PNIPAAm), which has been primarily utilized for biological applications.^18−20^ The benefit of using PNIPAAm in this area is its close-to-body-temperature LCST of approximately 32 °C that is easily tunable and relatively insensitive to variations in molecular weight or electrolyte concentration.^21,22^ However, limitations which arise from the use of PNIPAAm in this area include the presence of an evident hysteretic behavior when subjected to heating–cooling cycles,^23^ the high toxicity of its corresponding monomer,^24,25^ as well as the strong bio-adhesion with proteins by cooperative hydrogen bonding interactions.^18^ Hence, research interest has been lately devoted to identifying alternative LCST-type polymers to PNIPAAm.

One of the emerging classes involves polymers based on oligo(ethylene glycol) methyl ether methacrylate (OEGMA), which were widely recognized after the pioneering work of Lutz and co-workers in which they demonstrated the coil-to-globule phase transition of POEGMA-based polymers as well as the facile regulation of their LCST.^26−29^ To date, the phase transition behavior of such polymers has been widely explored, and it has been shown that the POEGMA side-chain groups are responsible for the solubility of the polymer in water and are thus controlling its LCST transition. When heated above the critical solution temperature, the ethylene glycol units undergo a thermo-reversible phase transition due to the disruption of hydrogen bonds formed between ethylene glycol and water molecules in favor of polymer–polymer interactions.^28,30−33^ Additional studies demonstrated that this phase separation could also lead to the formation of macromolecular aggregates or micelles depending on the composition and topology of the polymer chains.^34^ The LCST of these polymers can be accurately tuned upon selection of the right combination of short and long OEGMA side chains, each presenting different degrees of hydrophilicity. For instance, random copolymers of di(ethylene glycol) methyl ether methacrylate (DEGMA, *n* = 2) and poly(oligo(ethylene glycol) methyl ether methacrylate) (POEGMA, *n* = 8–9) exhibit LCST values between 26 and 90 °C, which can be precisely modified by varying the co-monomer composition.^26,29^ Over the past decades, reports related to OEGMA-based polymeric materials have grown rapidly in number due to several desirable properties such as their biocompatibility,^35,36^ the absence of hysteresis between heating and cooling cycles,^30^ and antifouling properties at temperatures below their LCST.^37,38^ Furthermore, the phase transition of OEGMA is relatively insensitive to important external parameters, such as the polymer concentration, the ionic strength of the media, as well as the polymer chain length.

The highly tunable and reversible aggregation behavior of POEGMA-based polymers is of particular interest for the development of thermoresponsive polymer-based systems that could be used for conformance control in enhanced oil recovery (EOR) applications. In oil recovery, poor sweep efficiency is one of the major issues that can reduce recovery factors by water flooding. More specifically, the injected flood water favors the path of least resistance and flows into regions of higher permeability (i.e., the thief zone) while bypassing less permeable zones, therefore missing oil in large areas of the reservoir, so the overall sweep is less efficient per barrel of water injected. A common strategy currently used to address the poor sweep efficacy involves the utilization of cross-linked polymers to permanently block thief zones in the subsurface, thereby diverting flow into previously unswept areas. Among polymers presently of interest for EOR, thermoresponsive polymers are useful because of a temperature differential encountered between the water injected into an oil reservoir and the reservoir itself, with the injected water generally being cooler than the connate fluids.^39,40^ Therefore, the exploitation of such temperature difference as a trigger to induce a change in the properties of the injected polymer solution once in the reservoir and to form a flow blockage has been also explored in order to reduce the permeability of the thief zone. Typically, the polymer and cross-linker are simultaneously injected into an oil reservoir to allow reaction upon reaching the high temperature front. However, this “*Deep Diverting Gel*” technology is limited by remaining amounts of the unreacted cross-linker and poor cross-linking efficiency as well as inefficient propagation of the formed gel far enough into the reservoir for successful blocking.^41^ Another polymer-based technology for EOR, termed *Bright Water*, utilized time-delayed, highly expandable polymer microparticles.^42,43^ The platform consists of highly cross-linked, sulfonate-containing polyacrylamide microparticles, which contain both labile and stable cross-links. The size of the particles has been optimized to allow injection without causing near wellbore damage, but upon heating, disruption of the labile crosslinking bonds occurs, resulting in concurrent particle swelling. The swollen particles were found to reduce the permeability of the thief zone and can effectively divert water into less permeable zones. However, this temperature-induced size increase is irreversible and, as such, the material is able to only form a single blockage within the oil reservoir that the injected aqueous solution will eventually circumvent, returning to the thief zone.^42,43^

The primary aim of this work was to develop thermoresponsive PDEGMA-based nanoparticles that could be used as flow modifiers within porous media, closely resembling the conditions and parameters of an oil reservoir. In this area, An and co-workers have reported the preparation of thermoresponsive core–shell polymer particles containing oligo(ethylene glycol) side chains by aqueous reversible addition–fragmentation chain transfer (RAFT) dispersion polymerization.^44^ This procedure led to the formation of small thermoresponsive particles with tunable size and enhanced stability in saline solutions over broad periods of time. As an example, this approach could be combined with the well-documented ability of POEGMA-based monomers to tune the LCST behavior of polymeric materials through copolymerization^26^ to produce a library of nanoparticles that possess variable responsive temperature. Herein, temperature-responsive and core-cross-linked P(DEGMA-*co*-OEGMA)-based block copolymer particles were synthesized via RAFT-mediated polymerization-induced self-assembly (PISA) in aqueous media. The resulting particles were found to display an LCST in aqueous media, which in turn led to a reversible aggregation behavior as a function of solution temperature. Upon varying the molar ratio of the OEGMA co-monomer within the core of the nanoparticles, the cloud-point and critical flocculation temperatures (*T*_CP_ and *T*_CFT_, respectively) of these particles were accurately regulated. In all cases, both *T*_CP_ and *T*_CFT_ were found to increase linearly with increasing core hydrophilicity (i.e., increasing mol % OEGMA), while experimental findings were also correlated to theoretical hydrophobicity calculations of oligomeric models. Through the use of an in-house sandpack rig, it was also demonstrated that these particles were able to modify the flow profile of porous media, producing stable and reversible blockages that could progress with the thermal front. Importantly, the depth of the blockage formation was found to strongly correlate with the responsive temperature of each particle formulation. To the best of our knowledge, this is the first report of thermoresponsive PDEGMA-based particles that act as programmable flow modifiers in porous media by reversible LCST-induced aggregation. The ability of these particles to modify the porous flow behavior sets the groundwork for the design of a highly efficient and viable polymer formulation technology, which could see its application be extended in EOR in future studies, as it has been the case for the *Bright Water* technology.^42,43^

## Results and Discussion

**Scheme 1:**
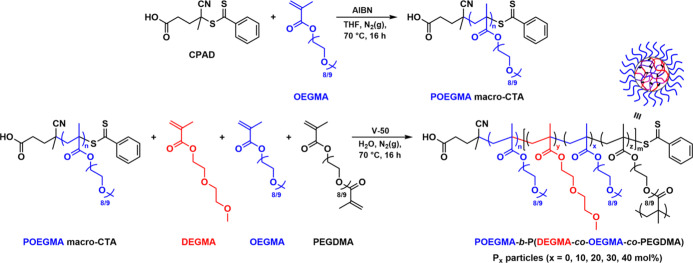
Reaction Scheme of the Synthetic Route Followed for the Preparation of POEGMA_50_ Macro-CTA via RAFT Polymerization and its Subsequent Chain-Extension Using DEGMA, OEGMA, and PEGDMA via RAFT-Mediated PISA to Form the Core Cross-Linked POEGMA_50_-*b*-P(DEGMA_*y*_-*co*-OEGMA_*x*_-*co*-PEGDMA_*z*_) **P**_***x***_ Nanoparticles

In order to produce P(DEGMA-*co*-OEGMA)-based block copolymer nano-objects, which display thermo-reversible behavior and tunable *T*_CP_ and *T*_CFT_ for their application as flow modifiers in porous media, our efforts focused on varying the molar ratio of PDEGMA (LCST ≈ 26 °C) and POEGMA (LCST ≈ 90 °C) components within their core-forming blocks to generate a library of particles with intermediate LCST values. Our synthetic approach was based on a modified version of the method reported by Shen et al. that describes the synthesis of thermo-sensitive nanoparticles containing a PDEGMA-rich core and a POEGMA-based shell.^44^ In particular, our investigation first involved the synthesis of a water-soluble macromolecular chain-transfer agent (macro-CTA) by reversible addition–fragmentation chain transfer (RAFT) polymerization. This was achieved via the homopolymerization of OEGMA using 4-cyano-4-(phenylcarbonothioylthio)pentanoic acid (CPAD) as the chain-transfer agent (CTA) and 2,2′-azobis(2-methylpropionitrile) (AIBN) as the radical initiator at 70 °C in THF for 16 h, targeting an average degree of polymerization (DP) of 50 (Scheme 1). The monomer conversion was monitored by ^1^H NMR spectroscopic analysis by comparing the relative integration of the OEGMA vinyl peaks (δ = 5.76 and 6.13 ppm) to the polymer backbone (δ = 2.24 ppm), and it was observed that the polymerization progressed to >99% total monomer conversion (*M*_n, NMR_ = 25.2 kDa). Following purification by dialysis and lyophilization, size-exclusion chromatography (SEC) analysis of the resulting POEGMA_50_ macro-CTA revealed the well-controlled character of the polymerization process, as judged by the monomodal and narrow molecular weight distribution and low *D̵* value of the obtained homopolymer (*M*_n, SEC_ = 17.5 kDa, *D̵* = 1.28) (Figure S2 and Table S1). Moreover, the near-complete overlap observed between refractive index and UV (λ = 309 nm) traces recorded by SEC confirmed the adequate retention of dithiobenzoate end-groups of the CTA on the homopolymer chains, making this POEGMA_50_ macro-CTA suitable for further chain-extensions via RAFT polymerization to create block copolymers.

The resulting POEGMA_50_ macro-CTA was then utilized as the hydrophilic steric stabilizer block for aqueous RAFT-mediated PISA using DEGMA and OEGMA as the core-forming monomers at varying molar ratios and poly(ethylene glycol) dimethacrylate (PEGDMA) (average *M*_n_ = 550) as the cross-linker (Scheme 1). Initiation of each PISA process was achieved using V-50, and all reactions were maintained at 70 °C for 16 h. This resulted in the formation of aqueous dispersions of cross-linked amphiphilic POEGMA_50_-*b*-P(DEGMA_*y*_-*co*-OEGMA_*x*_-*co*-PEGDMA_*z*_) (**P**_***x***_, *x* = 0, 10, 20, 30, 40 mol %) block copolymer nano-objects in situ. In total, a series of five particle formulations were prepared by varying the molar ratio of core-forming DEGMA and OEGMA monomers while targeting a constant average DP of 825 for the entire core block. The total monomer conversion was monitored by ^1^H NMR spectroscopic analysis by comparing the relative integration of the methacrylate vinyl peaks (δ = 5.7–6.2 ppm) to trioxane (δ = 5.17 ppm), which was used as an internal standard. In all cases, the polymerization progressed to >99% total monomer conversion. It should be noted that it was not possible to obtain molecular weight and dispersity information of the developed block copolymers due to their irreversibly cross-linked nature that makes them insoluble to common SEC solvents.

**Figure 1:**
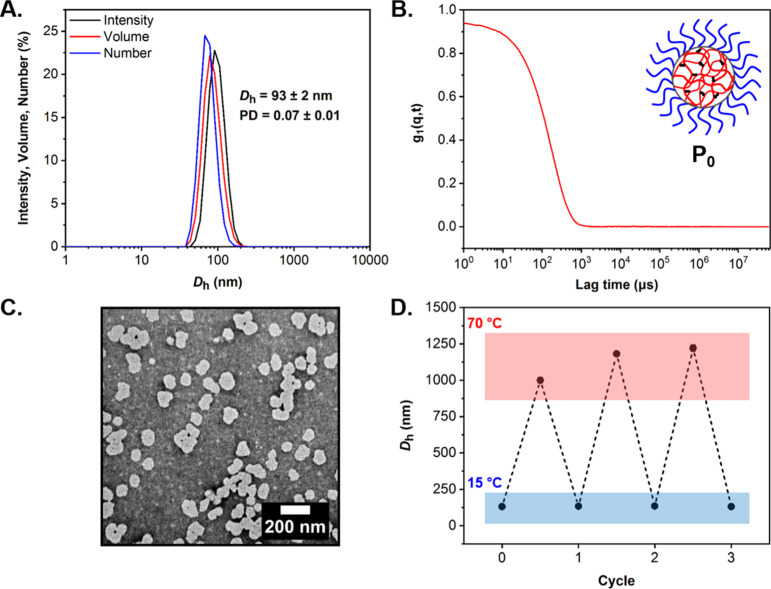
Characterization of cross-linked POEGMA_50_-*b*-P(DEGMA_825_-*co*-PEGDMA_12_) **P**_**0**_ particles. (A) DLS analysis showing the intensity-, volume-, and number-weighted size distributions along with average *D*_h_ and PD values (the error shows the standard deviation from four repeat measurements), (B) autocorrelation function as obtained by DLS, (C) representative dry-state TEM image, stained with 1 wt % uranyl acetate (UA) solution, and (D) reversible aggregation behavior for **P**_**0**_ particles reporting changes in *D*_h_ as a function of solution temperature (data was recorded over three heating–cooling cycles from 15 to 70 °C in a single step of 55 °C by variable temperature DLS analysis). All analysis was performed at a particle concentration of 1 mg mL^–1^ in 0.3 M NaCl_(aq)_.

The resulting **P**_***x***_ particles (where *x* denotes the molar ratio of POEGMA within the particle core relative to DEGMA) were characterized by dynamic light scattering (DLS) and dry-state transmission electron microscopy (TEM) in order to explore the effect of varying the molar ratio between the core-forming monomers on the size and morphology of the resulting core–shell particles (Figures 1 and S3–S8). The average hydrodynamic diameter (*D*_h_) and associated polydispersity (PD) values measured by DLS are compiled in Table 1. For the platform **P**_**0**_ particles containing 0 mol % of POEGMA, an average *D*_h_ of 93 ± 2 nm was measured with a corresponding PD of 0.07 ± 0.01. With the exception of **P**_**10**_ formulation, the *D*_h_ and PD correlated with the POEGMA content within the particle core, with the higher PD values indicating a broader size distribution. However, DLS characterization of nano-objects which possess a higher size distribution, that is, PD > 0.2, is less accurate due to the stronger scattering produced by larger particles or aggregates in the sample. This was also confirmed by the poor overlap between intensity-, volume-, and number-weighted distribution for **P**_**30**_ and **P**_**40**_ along with the autocorrelation function whereby the decay of the signal was not entirely exponential (Figure S3).

**Table 1:** Summary of P(DEGMA-*co*-OEGMA)-Based **P**_*x*_ Particles’ *D*_h_, PD, *D*_ave_, *T*_CP_, and *T*_CFT_ values

sample	DEGMA/OEGMA molar ratio	*D*_h_ (nm)^a^	PD^a^	*D*_ave_ (nm)^b^	*T*_CP_ (°C)^c^	*T*_CFT_ (°C)^d^
**P**_**0**_	100:0	93 ± 2	0.07 ± 0.01	95 ± 22	25	22
**P**_**10**_	90:10	144 ± 1	0.24 ± 0.01	66 ± 17	35	32
**P**_**20**_	80:20	71 ± 1	0.14 ± 0.01	56 ± 11	48	45
**P**_**30**_	70:30	29 ± 3	0.55 ± 0.08	51 ± 8	56	50
**P**_**40**_	60:40	20 ± 2	0.37 ± 0.01	164 ± 23	62	58

a Determined by DLS analysis.

b Determined by dry-state TEM imaging.

c Determined by UV–vis spectroscopy.

d Determined by variable-temperature DLS analysis.

Imaging of the aqueous **P**_***x***_ dispersions by dry-state TEM confirmed the presence of uniform populations of spherical particles regardless of the proportion of OEGMA co-monomer within their core (Figures 1C and S4–S8). The acquired TEM images were then used to produce histograms of particle size distributions and enable calculation of the average diameter (*D*_ave_) in each case (Figures S4–S8 and Table 1). It should be noted that there was no evident correlation between the *D*_ave_ and the molar ratio of co-monomer in the particle core. Overall, DLS and TEM characterization indicated the formation of spherical core–shell nanoparticles, while it was apparent that the control over the particle size distribution decreased when increasing the POEGMA content. This could be a result of the increased hydrophilicity of OEGMA relative to DEGMA, which in turn reduced the stability of the formed particles during the PISA process and led to an increase of their size distribution. In order to further improve the control of the self-assembly process, the synthesis could be performed at a higher temperature (i.e., above 90 °C, which is the LCST of POEGMA homopolymer) to reduce the solubility of the particle cores. However, this is a rather limiting approach given that the boiling point temperature of water is 100 °C at atmospheric pressure. Additionally, the thermo-reversible aggregation behavior of **P**_**0**_ particles was further evaluated by variable temperature DLS analysis using a single-step procedure over multiple heating–cooling cycles from 15 to 70 °C, whereby particle size values drastically increased to >1 μm on heating as a result of local flocculation and completely reversed back to their original *D*_h_ values on cooling (Figure 1D).

**Figure 2:**
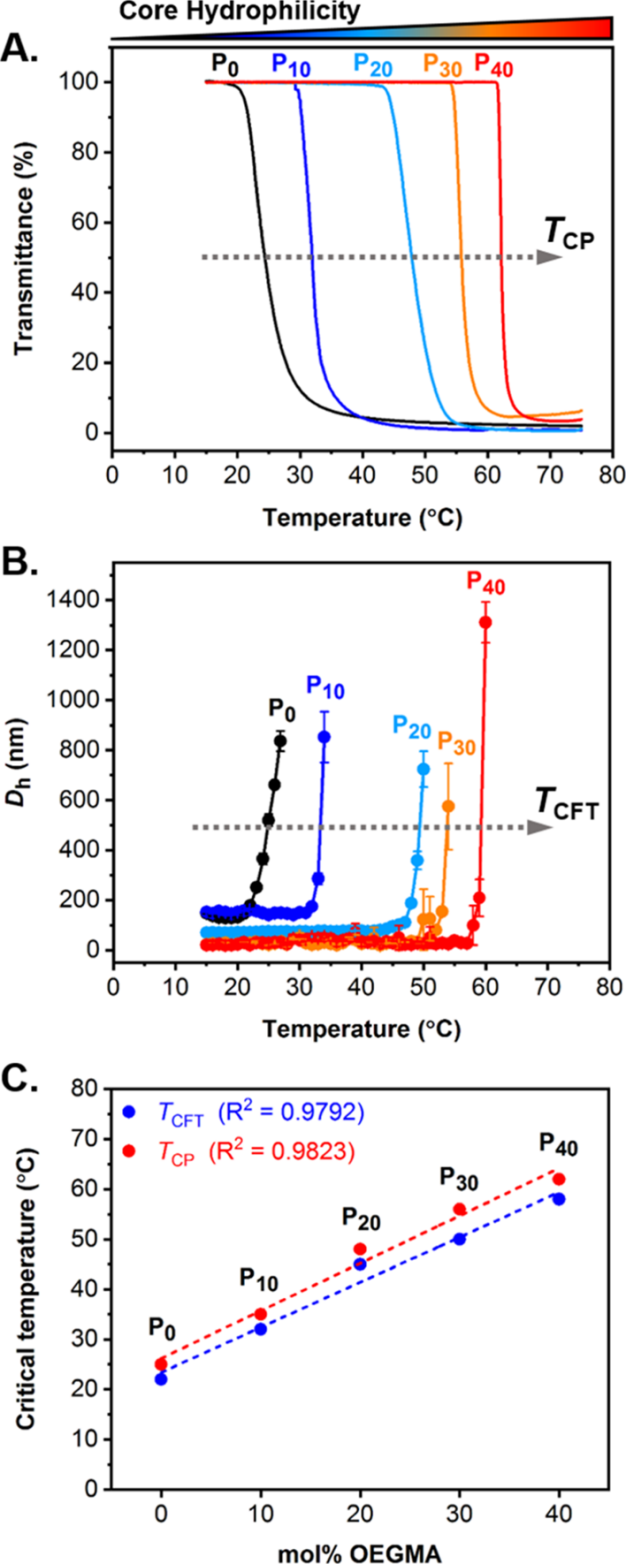
Evaluation of the thermoresponsive behavior of P(DEGMA-*co*-OEGMA)-based (**P**_***x***_) particles by (A) UV–vis spectroscopy and (B) variable temperature DLS analysis. UV–vis transmittance spectra were recorded at λ = 500 nm from 15 to 90 °C at 5 mg mL^–1^ in 0.3 M NaCl_(aq)_ and were used to determine *T*_CP_ values, while DLS analysis was performed from 15 to 90 °C at 1 mg mL^–1^ in 0.3 M NaCl_(aq)_ and was used to determine *T*_CFT_ values. (C) Correlation plot for measured *T*_CP_ and *T*_CFT_ values as a function of the mol % OEGMA content within the core of **P**_***x***_ particles.

The temperature-responsive nature of PDEGMA-based **P**_***x***_ particles was subsequently explored by variable-temperature UV–vis spectroscopy (Figure 2A) and DLS analysis (Figure 2B). During UV–vis spectroscopic analysis, the transmittance of aqueous **P**_***x***_ dispersions was recorded from 15 to 90 °C at a heating rate of 1 °C min^–1^ at a fixed wavelength of λ = 550 nm. For each formulation, the transmittance sharply decreased upon heating, also indicated by the aqueous solution becoming cloudier, as a result of particles becoming more solvent-insoluble when heated above their LCST. This phase transition was characterized by the *T*_CP_, recorded as the temperature at which half of the maximum transmittance occurred. Expectedly, *T*_CP_ values were shown to be directly related to the POEGMA content within the particle core, whereby they increased upon increasing mol % OEGMA (Figure 2A). Using variable-temperature DLS analysis (Figure 2B), the *D*_h_ of **P**_***x***_ dispersions was recorded from 15 to 90 °C in 1 °C intervals. For each formulation, the *D*_h_ significantly increased upon heating above a critical solution temperature, accompanied by the formation of larger aggregates as a result of the decreased solubility of particles when heated above their LCST. This is a well-established feature for thermoresponsive polymers and polymeric nano-objects that display an LCST-type phase transition.^12,45−47^ In each case, this phase transition temperature is reported here as the *T*_CFT_, recorded as the *D*_h_ value at the onset of exponential particle size increase. Similar to the trend observed for *T*_CP_ values, *T*_CFT_ increased with the growing hydrophilicity of particles when increasing the POEGMA content. The reproducibility of the *T*_CFT_ was explored by DLS on three different batches of **P**_**0**_ particles and showed an excellent repeatability (Figure S13). Both *T*_CP_ and *T*_CFT_ values measured for **P**_***x***_ particles are summarized in Table 1 and are also plotted against the molar ratio of POEGMA (Figure 2C). The obtained scatter graph clearly showed that both critical response temperatures increased linearly when increasing the POEGMA content. The *T*_CP_ and *T*_CFT_ were found to be fairly similar, with a constant offset observed between the two values for every particle formulation, which was most likely attributed to fundamental differences in the two characterization techniques. Indeed, *T*_CP_ was recorded at half-way during the observed phase transition, while each *T*_CFT_ was recorded at the start of the characteristic size transition. As such, this phenomenon could explain the constant gap observed between *T*_CP_ and *T*_CFT_ and why measured *T*_CFT_ values were always lower than *T*_CP_. Therefore, it was hypothesized that both phase transitions occurred almost simultaneously. Together, these findings provided evidence that the approach used herein allowed for fine tuning of both *T*_CP_ and *T*_CFT_ of P(DEGMA-*co*-OEGMA)-based particles by simply varying the molar ratio of core-forming monomers, but at the cost of reducing the achievable control over their size distribution when increasing particle core hydrophilicity.

**Figure 3:**
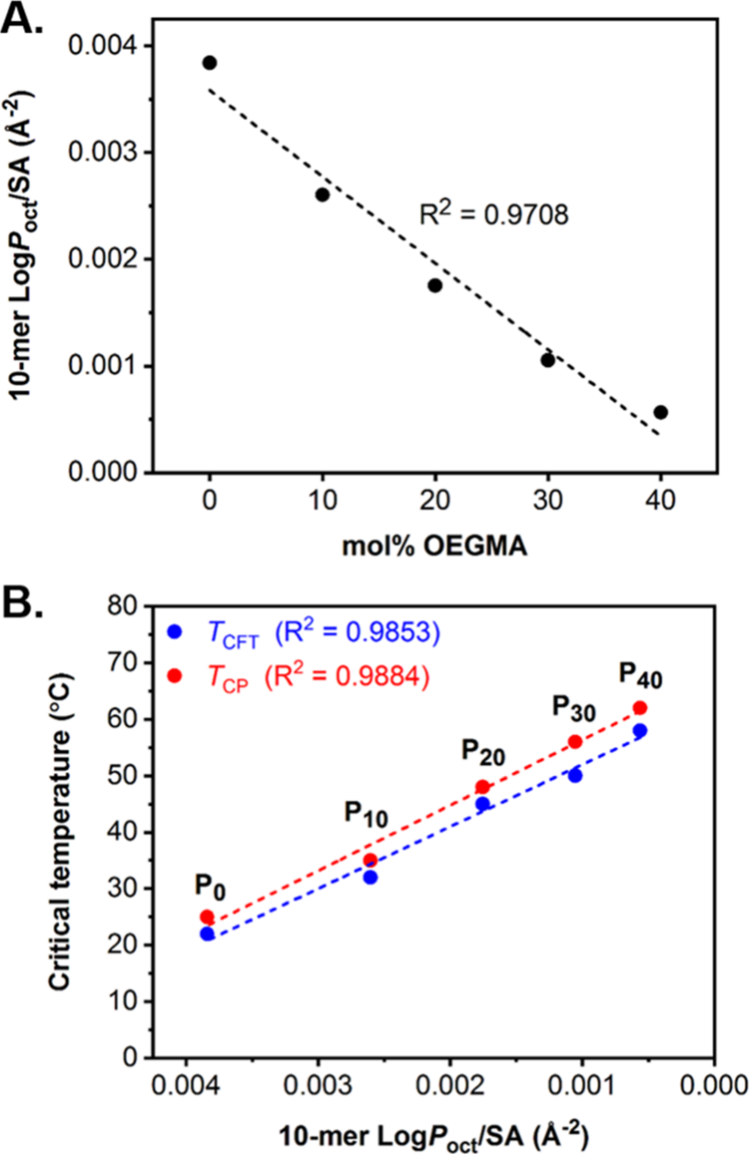
(A) Evolution of 10-mer hydrophobicity as a function of the mol % OEGMA content in the representative 10-meric model and (B) correlation of *T*_CP_ and *T*_CFT_ as a function of increasing 10-mer hydrophilicity (i.e., decreasing Log*P*_oct_/SA values). Log *P*_oct_ values (ALog*P* method) were calculated using an atom-based approach and normalized by solvent-accessible SA using Materials Studio 2020.

Next, we aimed to investigate the correlation between *T*_CP_ and *T*_CFT_ measured by UV–vis spectroscopy and DLS analysis, respectively, with the computationally calculated core hydrophilicity of the corresponding particles as a function of the relative molar ratio of the core-forming monomers. This theoretical model could then be used as a predictive tool to calculate the critical solution temperature of ab initio-designed particle formulations. The hydrophobicity/hydrophilicity of a molecule can be determined by calculating its partition coefficient Log *P*_oct_, which describes the distribution of a substance between an octanol-rich and water-rich region.^48^ In an attempt to minimize variability associated with the molecular weight of a polymer and end-group contribution when varying the molar ratio of the comprising co-monomers, Log *P*_oct_ values were normalized by solvent-accessible surface area (SA).^48−51^ Log *P*_oct_/SA values can either be positive or negative depending on the preference of the examined oligomer/polymer to partition either in the octanol or the water phase, respectively.^48,52−54^ The calculation of Log *P*_oct_/SA values can rapidly become computationally restrictive, especially for larger molecules such as polymers of large DPs, which require the use of powerful systems to perform such calculations. Therefore, Log *P*_oct_/SA values were calculated herein for short 10-meric P(DEGMA-*co*-OEGMA)-based models, resembling the core chemistry of developed **P**_***x***_ particles, containing variable DEGMA/OEGMA molar ratios and vinyl end-groups (Scheme S1). Taking into consideration that the position of each monomeric unit in the theoretical oligomeric models (blocky vs statistical oligomers) was found to not affect the calculated Log *P*_oct_/SA values, models based on blocky copolymers were used throughout our study. In this work, Log *P*_oct_/SA values were calculated using Materials Studio 2020 and plotted against the mol % OEGMA content in each representative oligomeric model (Figure 3A). Notably, it was found that Log *P*_oct_/SA values decreased with increasing the molar ratio of OEGMA in a linear manner. This gradual decrease of Log *P*_oct_/SA indicated that the 10-meric models became progressively more hydrophilic when increasing the mol % OEGMA content. Therefore, this theoretical investigation further supported our original hypothesis that the hydrophilicity within the particle cores increased with increasing molar ratio of OEGMA relative to DEGMA. Additional investigations revealed that both *T*_CP_ and *T*_CFT_ values measured for each **P**_***x***_ particle formulation increased linearly with decreasing Log *P*_oct_/SA values calculated for the corresponding 10-meric models (Figure 3B). As previously described, a similar linear correlation was observed when plotting responsive temperatures against the molar ratio of OEGMA_500_ in the particle core (Figure 2C). Overall, these findings indicated that the theoretical model used for Log *P*_oct_/SA calculations was accurate to quantify the core hydrophilicity of **P**_***x***_ particles and can be subsequently used as a computational tool to reliably predict the critical trigger temperatures of other thermoresponsive polymeric nanoparticles.

**Figure 4:**
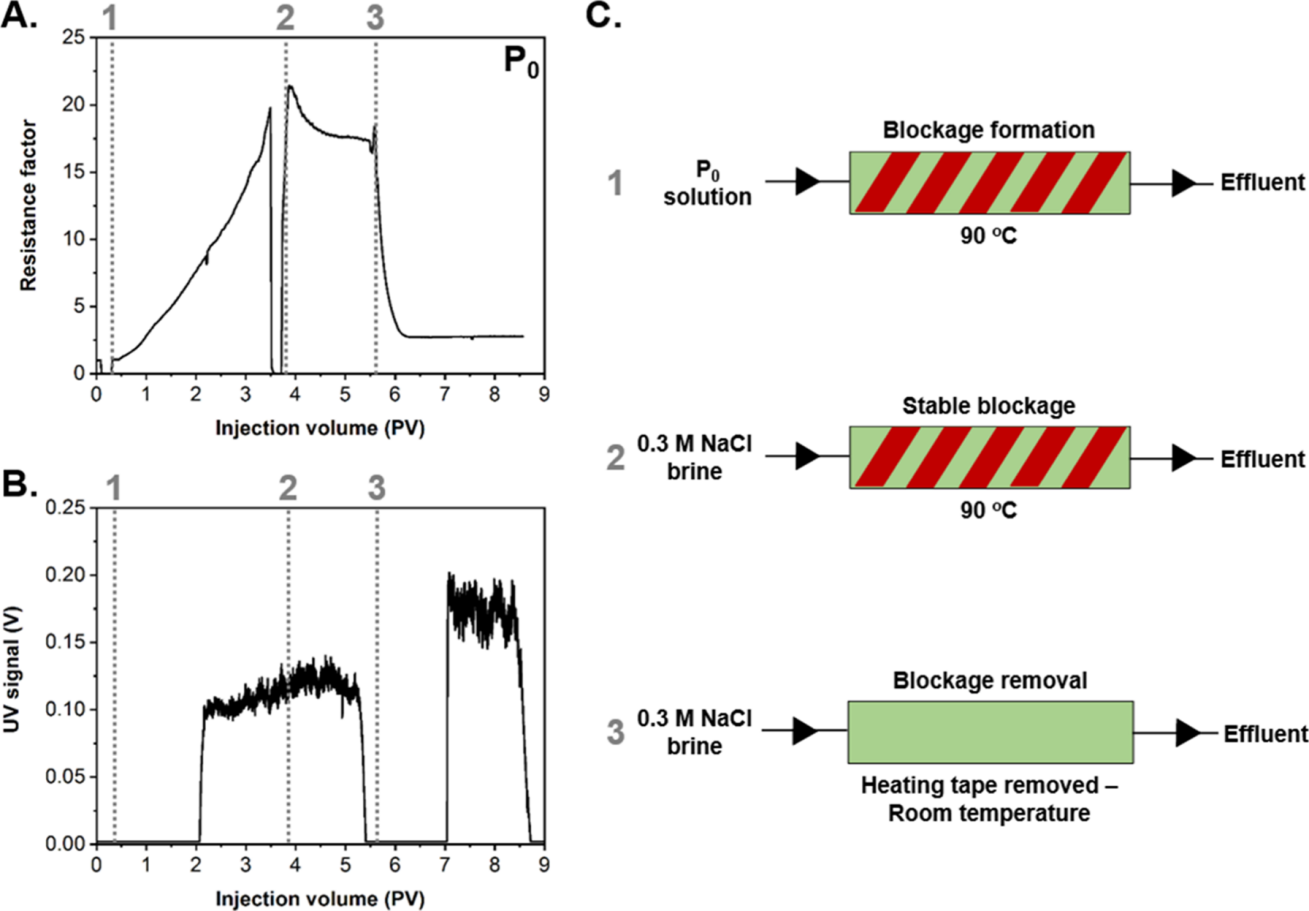
(A) Pressure drop profile of PDEGMA-based **P**_**0**_ particles containing *x* = 0 mol % OEGMA injected at 3 mg mL^–1^ (0.3 M NaCl, pH = 5.5, flow rate = 0.1 mL min^–1^). (B) Monitoring of the UV signal of the effluent during a single-column sandpack experiment. (C) Schematic representation of the three different stages and conditions for the performed single-column sandpack analysis.

The ability of the developed themoresponsive **P**_***x***_ block copolymer nano-objects to modify the flow of an aqueous solution within a porous medium was then evaluated using an in-house-built sandpack apparatus. This experiment consists of pumping a fluid through a column filled with sand and measuring the differential pressure (d*P*) produced across the column. In this case, the sand is equivalent to a porous medium that mimics the oil reservoir. A fluid characterized by lower mobility will produce a higher d*P* when flowing through the column.^55,56^ Polymer nano-particles in the effluent can be detected using a UV detector, but this measurement is only qualitative. In an initial experiment, a sample of **P**_**0**_ (3 mg mL^–1^ in 0.3 M NaCl_(aq)_) was injected at a constant flow rate of 0.1 mL min^–1^ into a column filled with sand (grain size = 45–65 μm) and pre-heated to 90 °C (Figure 4). The column permeability at 90 °C was 1.1 D and determined by measuring the differential pressure across the column and different flow rates and applying Darcy’s law. The data recorded in this experiment refer to monitoring of the d*P* across the sandpack column and the UV signal of the effluent, which were recorded against the volume of solution injected, normalized by the pore volume (PV) in the column. The measured pore volume in the packed column was 5 mL. The column was first saturated with 0.3 M NaCl at room temperature until a constant d*P* was recorded. Then, the column was heated to 90 °C and the **P**_**0**_ particle solution was injected, causing an incremental increase in d*P* with increasing pore volume of the solution injected (Figure 4A). It was hypothesized that the increase in the d*P* arose from the aggregation and precipitation of particles in the column when heated above their *T*_CFT_. The local particle flocculation progressively obstructed pores in the sandpack medium to form a blockage, which in turn generated the differential pressure increase recorded in the column. With no block formation, particles would be expected to exit the column after 1 PV, with full column saturation occurring after 2 PV. Following a period after the injection of **P**_**0**_, the UV signal increased, which indicated the presence of particles in the effluent—this delay in response suggests a partial block formation, implying that not all particles injected were retained in the column during the injection process (Figure 4B). After reaching a significant increase in d*P* (at least 10 times the baseline d*P*), the injection solution was switched to a 0.3 M NaCl solution with the aim of exploring the stability of the generated particle blockage. The injection of brine was performed at the same flow rate and column temperature (i.e., 90 °C). This caused an initial rapid decrease in d*P*, followed by a much slower decline, tending to a plateau. This suggested the initial flushing through of a small number of mobile particles and the formation of a largely stable blockage (Figure 4A). Monitoring of the UV signal further supported this, rising slightly and then stabilizing, suggesting that no further material was lost from the blockage over time (Figure 4B). The stable resistance factor (RF) and the absence of particles in the effluent provide evidence that the blockage formed by the flocculation of the PDEGMA-based particles was stable under the injection of brine as long as the column remained heated above their LCST.

Furthermore, aiming to dissipate the formed blockage while still injecting brine, heating of the column was stopped, and the column was allowed to progressively cool down to room temperature. The d*P* rapidly decreased, which correlates to the dissipation of the blockage by the re-dispersion of **P**_**0**_ particles in brine at lower temperatures (Figure 4A). Following the blockage dissipation, the UV signal was monitored to rise between 7 and 9 PV as re-dispersed particles were flushed from the column into the effluent (Figure 4B). The higher intensity of the UV signal for the dissipated blockage in comparison to the initial blockage formation indicated that the concentration of particles in the effluent was higher, suggesting that the accumulation of particles to build a pore blockage produced an increase of the local concentration of the particles. In this case, the d*P* did not return to its initial value due to the fact that the column was cooled at room temperature and, therefore, the solution injected was more viscous which, in turn, caused an increase in d*P*. The findings of this initial experiment provided clear support for the ability of **P**_**0**_ to form a stable and reversible blockage within porous media when heated above its *T*_CFT_. Importantly, we believe that the blockage formation and dissipation were driven by the LCST phase transition of **P**_**0**_ formulation, which led to a reversible aggregation/precipitation of the particles in solution when heated above their *T*_CFT_.

In the case of a field deployment, particles will be applied in contact with crude oil, which could potentially affect the particles’ properties. However, the stability of related particles in the presence of crude oil has been studied by one of our commercial partners for safety regulation limitation at the University of Birmingham. At this moment of time, those results cannot be disclosed, but it was observed that **P**_**0**_ particles were able to form a stable and reversible block with no alteration of their properties.

**Figure 5:**
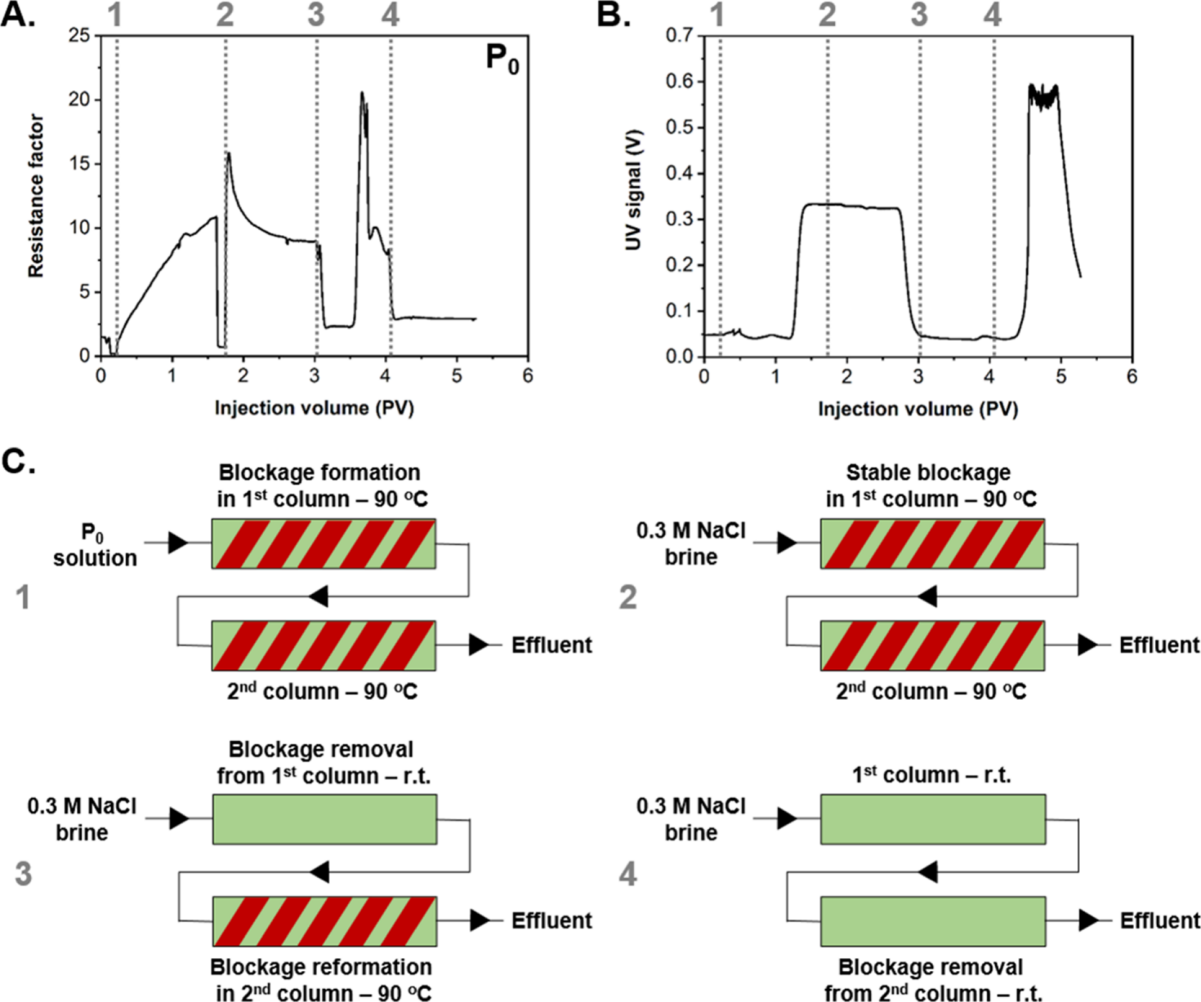
(A) Pressure drop profile of PDEGMA-based **P**_**0**_ particles containing *x* = 0 mol % OEGMA injected at 3 mg mL^–1^ (0.3 M NaCl, pH = 5.5, flow rate = 0.1 mL min^–1^). (B) Monitoring of the UV signal of the effluent during a double-column sandpack experiment. (C) Schematic representation of the four different stages and conditions for the performed double-column sandpack analysis.

For a field application, having validated the effectiveness of **P**_**0**_ nanostructures to generate a stable and reversible blockage in a porous medium by sandpack analysis, it was also of interest to investigate the ability of the blockage to move with the thermal front (i.e., the injected water front) along the thief zone to the producer, allowing sweep of the full reservoir without the requirement for multiple repeats. Experimentally, this was tested by attempting to form an initial blockage, clearing it and re-forming it in a later section of the apparatus. This was carried out using the previously described sandpack methodology, but with the addition of a second sand-filled column in series with the first one and the installation of a single differential pressure transducer across both columns (Figure 5). Initial results of this proof-of-concept double-column experiment were in good agreement with the single-column sandpack test previously reported. Indeed, the d*P* incrementally increased while injecting **P**_**0**_ into the first heated column, which indicated the aggregation of the particles and the formation of a partial blockage (again a small number of particles was detected in the effluent), which remained largely stable upon switching the injection to 0.3 M NaCl at 90 °C (Figure 5A,B). As there was only a single differential pressure transducer across both columns, it was not possible to accurately determine whether the blockage was solely formed in the first column or if there were multiple blocks. It was assumed there was a single blockage in the first column. At this stage of the experiment, brine was injected and the heating tape was removed from the first column to allow for the original blockage to clear while maintaining the heating tape on the second column. Consequently, the d*P* was shown to rapidly decrease, suggesting block dissipation. There was still a detectable baseline d*P* difference, which could suggest residual particles adsorbed to the first column (as shown in Figure 4A), but it may also indicate a secondary block in the second column. The plateau was followed up by a sharp increase of the d*P*, which could point to the breakdown of a block in the first column and a re-forming of the blockage on re-heating of the particles in the second column. With no further injection of **P**_**0**_ particles, the only polymer available is from breakdown of a primary block (Figure 5A). This sharp increase in d*P* at PV = 3.5 is consistent with the propagation time of particles from the first to the second column (each column contributing 0.5 to the total PV). However, if the d*P* rise is due to block reformation, then it happens much faster than with the primary block, suggesting some particle propagation and adsorption in the second column during the initial injection of **P**_**0**_. Alternatively, breakdown of the first block could lead to a higher local particle concentration than that of the stock solution in the original injection, leading to faster blocking. Separate differential transducers across each column would be required to definitively conclude blockage movement. Finally, the heating was removed from the second column and the d*P* decreased, which also confirmed the clearance of the blockage from the second column (Figure 5A). This was followed by an increase of the UV signal that indicated the presence of particles in the effluent and confirmed the complete blockage dissipation (Figure 5B). As observed for the single-column sandpack test, the higher UV signal for the dissipated blockage indicated the presence of a higher local concentration of **P**_**0**_ particles. Despite the fact that in an oil reservoir the temperature will not vary from 90 °C to room temperature, this double-column sandpack test supported the premise that the blockage formed by **P**_**0**_ is able to reversibly progress through porous media with the temperature front. The minimum temperature required to enable block dissipation either in our sandpack apparatus or in a real oil reservoir setting will primarily rely on the critical trigger temperature of the nano-object formulation. Therefore, the development of particles which possess higher *T*_CP_ and *T*_CFT_ would possibly enable identification of a suitable field candidate formulation.

**Figure 6:**
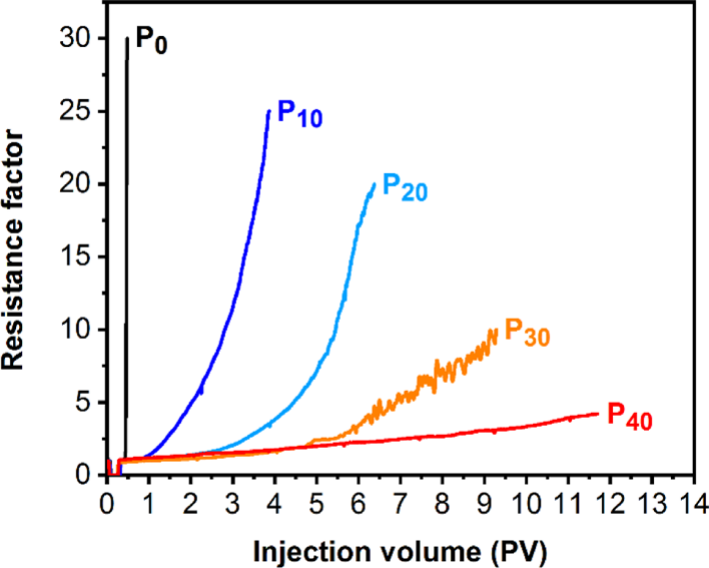
Evaluation of the pore-blocking performance of P(DEGMA-*co*-OEGMA)-based (**P**_***x***_) particles by sandpack analysis (5 mg mL^–1^, 0.3 M NaCl, pH = 5.5, flow rate = 0.1 mL min^–1^) upon monitoring changes in resistance factor (d*P*) with increasing injection volume (PV).

Consequently, to evaluate the effect of the responsive temperature of each particle formulation developed herein on the pore-blocking behavior, P(DEGMA-*co*-OEGMA)-based **P**_***x***_ particles were further characterized by single-column sandpack analyses (Figures S9–S12). Particle solutions were injected at 5 mg mL^–1^ in 0.3 M NaCl_(aq)_ at a flow rate of 0.1 mL^–1^ with the column pre-heated to 90 °C. In this case, injections were performed at higher concentrations due to the expectedly slower rate of blockage formation for particles with higher *T*_CFT_ values. Plots of the corresponding d*P* profiles showed that the rate of blockage formation strongly correlated with the responsive temperature of **P**_***x***_ particles (Figure 6). Indeed, results demonstrated that the rate of pore blocking decreased for particles with a higher responsive temperature due to the increased time (i.e., pore volume) required to exceed their critical solution temperature. For these experiments, all samples were injected from room temperature in a packed column heated at 90 °C and, therefore, were subjected to the same heating rate inside the column. However, these particles were shown to possess different critical solution temperatures and be subject to varying kinetics, which, for a given flow rate and column dimensions, would be reached after spending different amounts of time in the column. Hence, particles with a higher responsive temperature have slower kinetics and are prone to trigger and generate a stable blockage after spending more time in the column and, thus, position further along it. This can be advantageous in EOR applications for tuning the depth at which the blockage formation is triggered to ensure that it is deep enough in the reservoir to optimize placement and avoid generation of undesirable blockages in the near wellbore.

Regarding the differences in the observable blocking rate behavior, we speculated that particles with more hydrophilic cores are retained less by the column during the primitive stages of blockage formation. This behavior could also be accentuated by their delayed aggregation, which may cause the blockage to be more spread out over the column length and, thus, the local concentration of particles may be lower for the more solvent-accessible particles. Collectively, our results indicate that varying the trigger temperature of the injected particles drastically affects the pore-blocking kinetics. The rate of blockage formation could, accordingly, be adjusted by varying the concentration of injected particles, as observed by comparing the blocking profile of **P**_**0**_ particles at conc. = 3 mg mL^–1^ (Figure 4A) and 5 mg mL^–1^ (Figures 6 and S9).

Ultimately, with the only exception of **P**_**0**_, the d*P* and associated blockage generated remained stable under the subsequent injection of a 0.3 M NaCl solution and dissipated once the column was cooled down to room temperature. The incorporation of POEGMA within the particle cores and the higher responsive temperatures associated with a higher core hydrophilicity were, therefore, not detrimental for the blockage stability and reversibility, as judged by sandpack analysis. The blockage generated using **P**_**0**_ at 5 mg mL^–1^ did not entirely dissipate once the column was cooled down to room temperature (Figure S9). This was surprising taking into consideration that **P**_**0**_ was shown to reversibly dissipate at 3 mg mL^–1^. As such, it was hypothesized that the lack of reversibility observed for **P**_**0**_ at 5 mg mL^–1^, resulted from the high internal pressure generated by the blockage (RF ∼ 50) (Figure S9A). This could further compress the formed blockage and force it closer to the sand grains, increasing the particle adhesion. In order for a blockage to fully dissipate, there needs to be minor flow and agitation of the surrounding solution. In the event that the blockage is too strong, the particles could then be irreversibly adsorbed into the sand. Therefore, this can be considered as a limitation of a potential high-pressure setup rather than a lack of reversibility when injecting **P**_**0**_ at higher concentrations. Additionally, monitoring of the UV signal provides evidence of this hypothesis since a detectable population of particles was observed in the effluent once the column was cooled down, which revealed a partial re-dispersion of the aggregated particles.

During the course of our investigation, no stability issues were observed for particles held at high temperatures over prolonged periods of time. Indeed, the particle-induced blockage remained stable during single-column sandpack studies for **P**_***x***_ particles but also for the double-column sandpack test as shown for **P**_**0**_ particles. In addition, the excellent size reversibility for **P**_**0**_ particles was shown over three heating and cooling cycles by DLS. However, given the prolonged times of injection typically encountered in a EOR field application, future studies would focus on the longer term stability of our formulations at variable temperature, pH, and salinity environments.

## Conclusions

In summary, the synthesis of amphiphilic cross-linked P(DEGMA-*co*-OEGMA)-based nanoparticles by aqueous RAFT-mediated PISA is reported herein using a POEGMA macro-CTA as the corona-forming block. The developed library of nano-objects demonstrated thermo-reversible aggregation behavior driven by an LCST-type transition with increasing solution temperature. Furthermore, both *T*_CP_ and *T*_CFT_ values of the particles could be precisely modified as they were evidently shown to increase with increasing molar ratio of OEGMA within the particle core in a linearly dependent manner. Computational hydrophobicity calculations of 10-meric P(DEGMA-*co*-OEGMA)-based models were used to predictably correlate the effect of increasing core hydrophilicity on the corresponding critical transition temperatures. Sandpack analysis revealed that such core–shell particles could serve as flow modifiers in porous media. The particles were able to generate highly stable and reversible blockages that could progress with the thermal front. Notably, the effect of the responsive temperature on the pore-blocking performance was then further explored, and it was shown that reduction in blocking kinetics scaled proportionally with increasing both *T*_CP_ and *T*_CFT_ and core hydrophilicity of the injected particle formulations, positioning the blockage further along the sandpack. Interestingly, thermoresponsive particles with higher degrees of core hydrophilicity and *T*_CP_ values could still achieve a stable blockage while maintaining their reversible behavior as long as the concentration of injected particles was sufficiently high and the column was heated above both *T*_CP_ and *T*_CFT_. Ultimately, this procedurally simple and versatile approach for the development of thermoresponsive nanostructures with programmable flow behavior in porous media is expected to serve as a guide in future studies aiming toward the upscale and commercialization of a polymer particle technology for its application in EOR in an industrially relevant setting.

## Experimental Section

### Synthesis of POEGMA Macro-CTA

CPAD CTA (0.082 g, 0.30 mmol, 1.0 equiv), OEGMA (7.4 g, 15 mmol, 50 equiv) and AIBN (0.012 g, 0.073 mmol, 0.24 equiv) were added in a 50 mL round-bottom flask equipped with an oval-shaped stirring bar. THF (15 mL) was added, and the mixture was stirred at 250 rpm until a clear solution was obtained. The solution was then degassed by purging with N_2_(g) for 30 min under stirring at 250 rpm. The flask was then sealed and placed into a preheated oil bath set at 70 °C under continuous stirring at 250 rpm, and polymerization was allowed to proceed for 16 h to ensure full monomer conversion. After 16 h, the polymerization reaction was quenched upon cooling to room temperature and exposing to air. The conversion was monitored through ^1^H NMR spectroscopic analysis by comparing the relative integration of the methacrylate vinyl peaks to the polymer backbone, and it was observed that the polymerization progressed to >99% monomer conversion. The resulting POEGMA_50_ macro-CTA was purified by extensive dialysis against deionized water (MWCO = 1 kDa) and was recovered as a pink viscous liquid after lyophilization. The purified polymer was then characterized by ^1^H NMR spectroscopy and aqueous SEC analysis (Figure S2 and Table S1). ^1^H NMR (300 MHz, D_2_O) conv. >99%, *M*_n, NMR_ = 25,200 g mol^–1^. SEC (THF + 2% v/v NEt_3_) *M*_n, SEC_ = 17,500 g mol^–1^, *M*_w, SEC_ = 22,400 g mol^–1^, *D̵* = 1.28.

### Synthesis of Cross-Linked POEGMA-*b*-P(DEGMA-*co*-OEGMA-*co*-PEGDMA) Particles (**P**_***x***_) via RAFT-Mediated PISA Using POEGMA_50_ Macro-CTA as the Steric Stabilizer

The RAFT copolymerization of DEGMA, OEGMA, and PEGDMA using POEGMA_50_ macro-CTA as the steric stabilizer was performed in water at 70 °C using V-50 as the radical initiator. The molar ratios of macro-CTA, core-forming monomers, cross-linker, and radical initiator were kept constant (1:825:12:3.3), while the molar ratio between DEGMA and OEGMA within the core-forming block was varied. A general procedure for the synthesis of P(DEGMA-*co*-OEGMA)-based (**P**_***x***_) particles was as follows:

POEGMA_50_ macro-CTA (*M*_n, NMR_ = 25,200 g mol^–1^) (0.096 g, 0.0043 mmol, 1 equiv), DEGMA (0.54 g, 2.8 mmol, 660 equiv), OEGMA (0.35 g, 0.7 mmol, 165 equiv), and PEGDMA cross-linker (0.029 g, 0.053 mmol, 12 equiv) were dispersed in deionized water (50 mL) by stirring at 600 rpm. The resulting mixture was transferred to a polymerization ampoule, sealed, and purged with N_2_(*g*) for 30 min under stirring at 600 rpm and then heated for 30 min in an oil bath preheated at a temperature of 70 °C. The radical initiator V-50 (0.0039 g, 0.014 mmol, 3.3 equiv) was dissolved separately in deionized water (1 mL) and purged with N_2_(g) for 10 min. The degassed V-50 solution was added to the degassed polymerization solution to initiate the reaction. The polymerization mixture was then stirred at 600 rpm and at 70 °C for 16 h to ensure full monomer conversion. After 16 h, the polymerization reaction was quenched upon cooling to room temperature and exposing to air. This procedure resulted in formation of an aqueous dispersion of **P**_**20**_ particles in situ via RAFT-mediated PISA. The conversion was monitored by ^1^H NMR (300 MHz, D_2_O) for all polymerization reactions and was found to be >99% in all cases. The resulting particles in each case were further analyzed by DLS, UV–vis spectroscopy, and dry-state TEM imaging.

## Supporting Information Available

Materials and characterization techniques, oligomer hydrophobicity evaluation details, NMR and SEC analysis data for POEGMA macro-CTA, DLS analysis and TEM imaging data for P(DEGMA-*co*-OEGMA)-based **P**_***x***_ particles, and sandpack analysis data for P(DEGMA-*co*-OEGMA)-based **P**_***x***_ particles (PDF)
