# Supplementary material for: Thermoresponsive Block Copolymer Core–Shell Nanoparticles with Tunable Flow Behavior in Porous Media
Source: ACS Appl Mater Interfaces. 2022 Nov 19;14(48):54182–93. doi: 10.1021/acsami.2c15024 (PMC9743085; doi:10.1021/acsami.2c15024)
Supplement: Supplementary file 1 — am2c15024_si_001.pdf [file am2c15024_si_001.pdf]

Supporting Information

Thermoresponsive Block Copolymer Core-Shell Nanoparticles with Tunable Flow Behavior in Porous Media

*Matthieu P. J. Miclotte,[a] Spyridon Varlas,[a] Carl D. Reynolds,[a] Bilal Rashid,[b] Emma Chapman,[b] and Rachel K. O'Reilly[a,]**

[a] School of Chemistry, University of Birmingham, Edgbaston, Birmingham, B15 2TT, UK

[b] BP Exploration Operating Company Ltd., Sunbury-on-Thames, Middlesex, TW16 7LN, UK

**Corresponding Author*: r.oreilly@bham.ac.uk (R.K.O.R.)

Contents

Materials S3

Characterization techniques S3

Evaluation of oligomer hydrophobicity S7

Characterization data for poly(oligo(ethylene glycol) methyl ether methacrylate) (POEGMA) macro-CTA S9

DLS analysis of P(DEGMA-*co*-OEGMA)-based (**P_x**) particles containing x = 0, 10, 20, 30 and 40 mol% OEGMA within their core S10

Representative dry-state TEM images of **P_x** particles containing x = 0, 10, 20, 30 and 40 mol% OEGMA within their core S11

Sandpack analysis of **P_x** particles containing x = 0, 10, 20, 30 and 40 mol% OEGMA at 5 mg mL^{-1} in 0.3 M $NaCl_{(aq)}$ S14

Variable-temperature DLS analysis of different batches of **P_0** particles S18

Materials

2,2′-Azobis(2-methylpropionamidine) dihydrochloride (V-50) (97%), 4-cyano-4-(phenylcarbonothioylthio)pentanoic acid (CPAD) (≥99%), chloroform-*d* ($CDCl_3$) (≥99.8 atom % D), deuterium oxide (D_2O) (99.9 atom % D), hydrochloric acid (HCl) (ACS reagent, 37%), potassium persulfate (KPS) (≥99.0%), ProClin™ 300, sodium chloride (NaCl) (≥99.0%), sodium iodide (NaI) (≥99.5%) and sodium hydroxide (NaOH) (≥98%, anhydrous) were obtained from Sigma-Aldrich and were used as received. Di(ethylene glycol) methyl ether methacrylate (DEGMA) (95%), poly(ethylene glycol) dimethacrylate (average M_n = 550, PEGDMA) (99%) and oligo(ethylene glycol) methyl ether methacrylate (average M_n = 500, OEGMA) (99%) were also obtained from Sigma-Aldrich and were passed through a column of neutral aluminium oxide (Al_2O_3) for inhibitor removal prior to use. Tetrahydrofuran (THF) and Spectrum Spectra/Por dialysis tubing (MWCO = 1 kDa) were supplied by Fisher Scientific. 2,2′-Azobis(2-methylpropionitrile) (AIBN) (98%) was obtained from Sigma-Aldrich and was recrystallized from methanol. Aluminium oxide (activated, neutral, Al_2O_3) was obtained from Alfa Aesar and was used as received. Deionized water was obtained from a Triple Red Alto purification system and was used in all experiments unless stated otherwise. Brine was prepared by dissolving NaCl (17.62 g for 0.3 M) in 1 L deionized water and stirred for ~1 hour to ensure dissolution followed by addition of 0.05% v/v ProClin™ 300 (biocide). The solution pH was then adjusted to pH = 5.5 using either HCl 0.1 M or NaOH 0.1 M.

Characterization techniques

NMR Spectroscopy. ^{1}H-Nuclear magnetic resonance (NMR) spectra were acquired using a Bruker DPX-300 spectrometer using deuterium oxide (D_2O) or chloroform-*d* ($CDCl_3$) as the solvent at

room temperature. At least 32 scans were recorded for each sample and chemical shifts are reported as δ in parts per million (ppm) and are relative to either the solvent residual peak (HDO, δ = 4.79 ppm) when using D_2O or tetramethylsilane (TMS) at δ = 0 ppm when using $CDCl_3$.

Size Exclusion Chromatography. Size exclusion chromatography (SEC) analysis was performed on an Agilent 1260 Infinity II LC system equipped with an Agilent guard column (PLGel 5 μM, 50 × 7.5 mm) and two Agilent Mixed-C columns (PLGel 5 μM, 300 × 7.5 mm). The mobile phase used was THF (HPLC grade) containing 2% v/v NEt_3 at 40 °C at flow rate of 1.0 mL min^{-1} (polystyrene (PS) standards were used for calibration). Detection was conducted using a differential refractive index (RI) detector and an ultraviolet (UV) detector set to λ = 309 nm. Number-average molecular weights (M_n), weight-average molecular weights (M_w) and dispersities ($Đ = M_w/M_n$) were determined using the Agilent GPC/SEC software.

Dynamic Light Scattering. Dynamic light scattering (DLS) experiments were performed using either a Malvern Zetasizer Nano S or ZSP system equipped with a 633 nm He-Ne laser at either 4 mW or 10 mW, respectively. All size measurements were made using samples of concentration 1 mg mL^{-1} in 0.3 M $NaCl_{(aq)}$ at 15 °C, with light scattering detected at an angle of 173° (back-scattering). Hydrodynamic diameters (D_h) were determined using the Stokes-Einstein equation, which assumes perfectly monodisperse non-interacting spheres, and averaged over 4 consecutive runs with at least 10 measurements recorded for each run. Variable-temperature DLS analysis was performed from 15–90 °C in 1 °C intervals, with the sample equilibrated at the set temperature for 300s. Samples were prepared at a concentration of 1 mg mL^{-1} in 0.3 M $NaCl_{(aq)}$.

UV-Vis Spectroscopy. UV-Vis spectroscopy was performed using a Thermo Scientific Evolution 350 UV-Vis spectrophotometer equipped with a Xenon flash lamp light source and a dual-matched silicon photodiode detector. Quartz cells (170–2000 nm) from Hellma with two polished sides

were used for examining the transmittance spectral data by using Thermo INSIGHT-2 v.10.0.30319.1 software. A thermostat and 8-cell Peltier system with precise temperature control between 0 °C and 90 °C were coupled with Evolution 350 UV-Vis spectrophotometer to record T_{CP} values by recording temperature-dependent transmittance spectra of each sample from 15–90 °C at a heating rate of 1 ºC min^{-1} (λ = 550 nm). Samples were prepared at a concentration of 5 mg mL^{-1} in 0.3 M $NaCl_{(aq)}$.

Transmission Electron Microscopy. Dry-state stained transmission electron microscopy (TEM) imaging was performed on a JEOL JEM-1400 microscope at an acceleration voltage of 80 kV. All samples were diluted with 0.3 M $NaCl_{(aq)}$ to appropriate analysis concentration and then deposited onto formvar-coated copper grids. After approximately 1 min, excess sample was blotted from the grid and the grid was stained using an aqueous 1 wt% uranyl acetate (UA) solution for 1 min prior to blotting, drying and microscopic analysis. Average particle diameters (D_{ave}) were determined by measuring at least 100 particles per sample using the ImageJ software.

pH Measurements. pH Measurements were performed using a Mettler Toledo G20 compact titrator equipped with a DGi115-SC pH-electrode. The calibration of the glass pH-electrode was performed using buffer solutions at pH = 4.01, 7.00, and 9.21, obtained from Mettler Toledo. All data were recorded using the LabX Light Titration 3.1.1.0 software provided by Mettler Toledo.

Sandpack Analysis. Sandpack testing was performed on an in-house built system (**Figure S1**). The custom-made sandpack apparatus was set up to simulate linear flow and to study polymer pore blocking experiments. A glass column of 35 cm length and 6.6 mm internal diameter was filled with sand (45 – 65 μm). The column was saturated with solvent using a Strata DCP50 pump. Pressure drop across the column was measured with a differential pressure transducer and the permeability of the sandpack was calculated using Darcy's law. The mobile phase used was 0.3 M

NaCl brine adjusted to pH = 5.5 containing 0.5% ProClin™ 300 biocide. During each experiment, a flow rate of 0.1 mL min^{-1} was maintained and temperature was set to 90 ºC using heating tape around the column. The column was pre-heated prior to every sample injection. The eluent was monitored by UV (λ = 290 nm). All pieces of equipment were connected using 1 mm ID, 1.6 mm OD tubing. To measure the pore volume, NaI was dissolved in water to make a 0.01 mg mL^{-1} solution. The injection loop was filled with 2 mL NaI solution using a syringe. The injection loop was filled with 2-3 times the injection loop volume. The pump was run for enough time to ensure stabilization of the pressure and RI/UV detector. Then, the injection valve was switched on allowing for passage of the NaI solution into the column and the time was recorded from that moment by concurrent monitoring of the UV signal. The time between the injection and the signal increase multiplied by the flow rate gave the corresponding pore volume. This process was also repeated once without the column to subtract the contribution of the rest of the system.

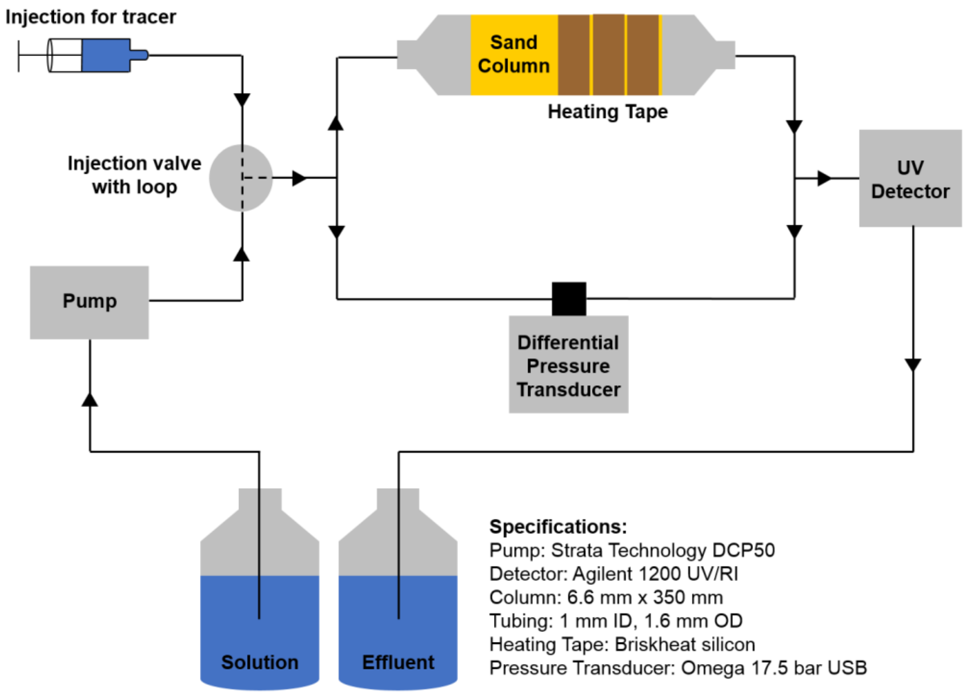

Figure S1: Schematic representation of the custom-made sandpack apparatus used in this study.

Evaluation of oligomer hydrophobicity

LogP_{oct} Analysis. Octanol-water partition coefficients (LogP_{oct}) were calculated for oligomeric models (10-mers) in Materials Studio 2020, using an atom-based approach (ALogP method) for all molecular models containing C, H, and O atoms.

Surface Area Analysis. Octanol-water partition coefficients (LogP_{oct}) were normalized by solvent accessible surface area (SA) using Materials Studio 2020. First, oligomers were subjected to a Geometry Optimization procedure using the Forcite Molecular Dynamics (MD) module with a COMPASS II force field. The force field contains information on important parameters, like preferred bond lengths, bond angles, torsion angles, partial charges, and van der Waals radii that influence the conformation. To minimize energy and determine a preferred conformation, these simulations ran until the energy of the oligomer decreased below predetermined convergence criteria (1×10^{-4} kcal mol^{-1} energy convergence, 0.005 kcal mol^{-1}/Å force convergence, and 5×10^{-5} Å displacement convergence). Second, these SA values represent solvent accessible surface area created by an algorithm that rolls a ball over the surface of the oligomer. To ensure the SA values are meaningful in the context of octanol-water partition coefficients (LogP_{oct}), the probe had a 1.4 Å radius to match the size of a water molecule.

Models. Scheme S1 depicts a representative example of P(DEGMA-*co*-OEGMA)-based ($\mathbf{P_x}$) 10-mers containing x = 0, 10, 20, 30 and 40 mol% of OEGMA units. To simplify calculations, blocky oligomers with a consistent *cis* conformation were selected for analysis in all cases (an all-*trans* conformation did not affect calculated LogP_{oct}/SA values).

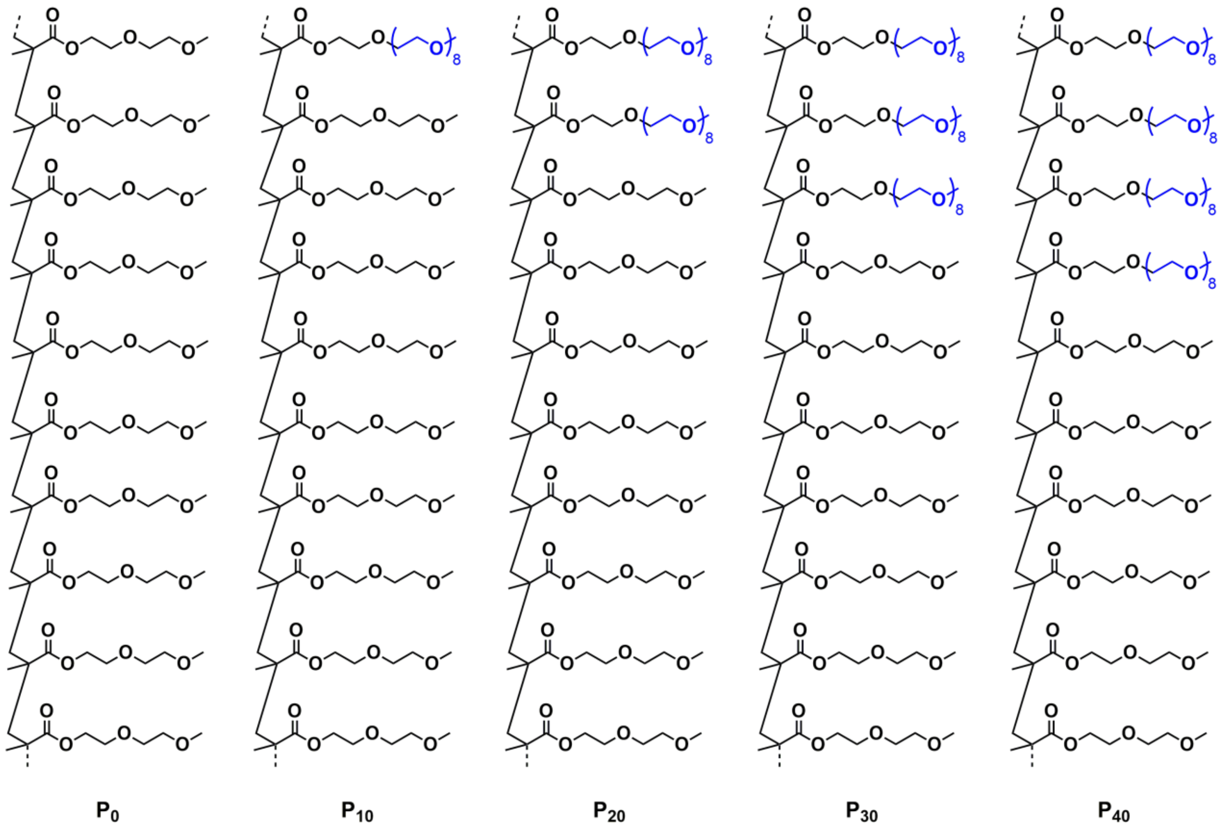

Scheme S1: Representative structures of P(DEGMA-*co*-OEGMA)-based (**P$_x$**) 10-mers containing x = 0, 10, 20, 30 and 40 mol% of OEGMA units used for LogP_{oct} calculations.

Characterization data for poly(oligo(ethylene glycol) methyl ether methacrylate) (POEGMA) macro-CTA

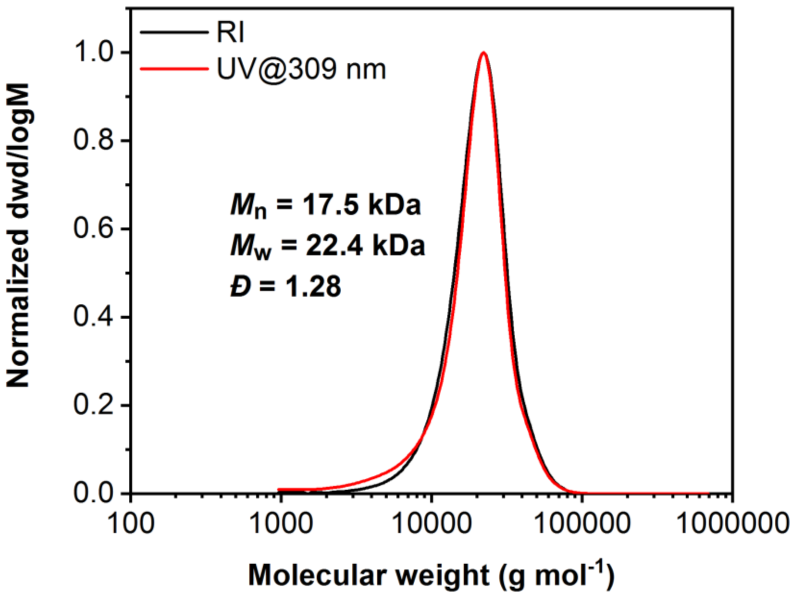

Figure S2: Normalized SEC RI (black trace) and SEC UV (red trace, λ = 309 nm) molecular weight distributions for $POEGMA_{50}$ macro-CTA, along with corresponding M_n, M_w, and $Đ$ values calculated based on PS standards using THF + 2% v/v NEt_3 as the eluent.

Table S1: Molecular characteristics of $POEGMA_{50}$ macro-CTA, as determined by ^{1}H-NMR spectroscopy and SEC analysis.

[OEGMA]/[CPAD]	% Conv.[a]	$M_{n, theo.}$ (kDa)[b]	$M_{n, NMR}$ (kDa)[c]	$M_{n, SEC}$ (kDa)[d]	$Đ_{, SEC}$[d]
**50**	>99	25.3	25.2	17.5	1.28

[a]Monomer conversion calculated from ^{1}H-NMR spectroscopy in D_2O. [b]Calculated from conversion. [c]Calculated using end-group analysis from ^{1}H-NMR spectroscopy. [d]M_n and $Đ$ values calculated from PS standards using THF + 2% v/v NEt_3 as the eluent.

DLS analysis of P(DEGMA-*co*-OEGMA)-based (P_x) particles containing x = 0, 10, 20, 30 and 40 mol% OEGMA within their core

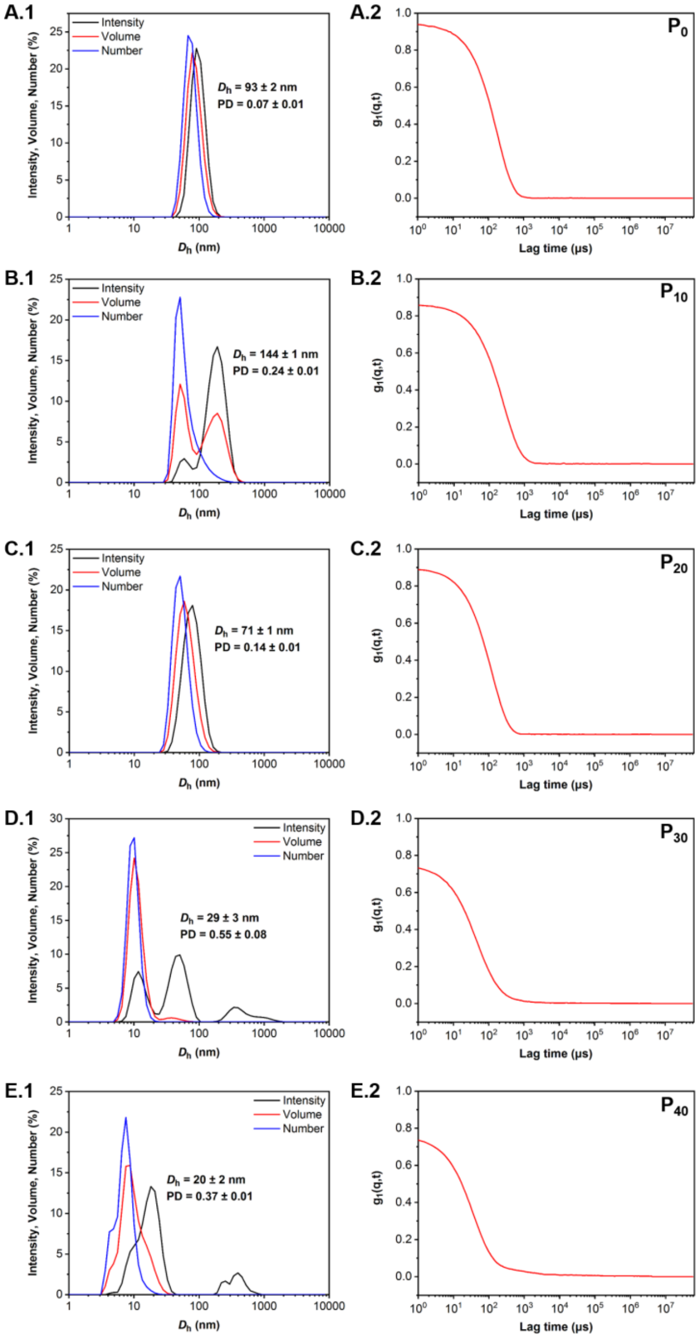

Figure S3: (1) Size distributions along with average D_h and PD values (from 4 repeat measurements) and (2) corresponding correlation function, obtained by DLS analysis, for $\mathbf{P_x}$ particles containing (A) 0 mol%, (B) 10 mol%, (C) 20 mol%, (D) 30 mol% and (E) 40 mol% of OEGMA. Recorded at 15 °C, conc. = 1 mg mL^{-1} in 0.3 M NaCl solution.

Representative dry-state TEM images of P_x particles containing x = 0, 10, 20, 30 and 40 mol% OEGMA within their core

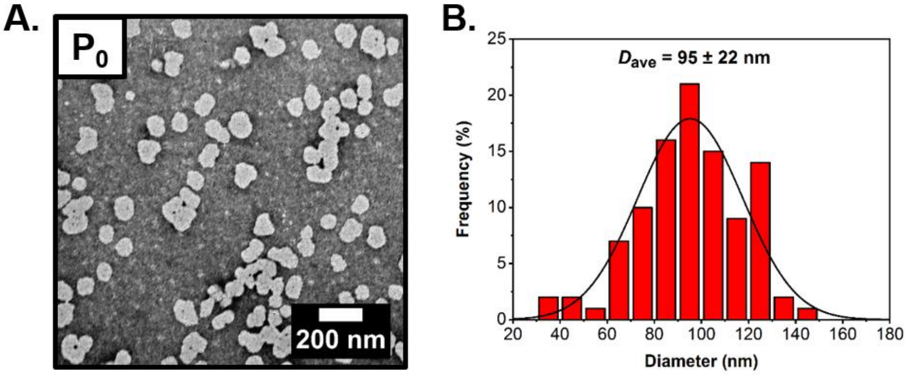

Figure S4: (A) Representative dry-state TEM image of PDEGMA-based $\mathbf{P_0}$ particles containing x = 0 mol% OEGMA, stained with 1 wt% uranyl acetate (UA) solution (scale bar represents 200 nm), and (B) histogram of their corresponding size distribution along with calculated average diameter, measured from particle analysis based on acquired dry-state TEM images.

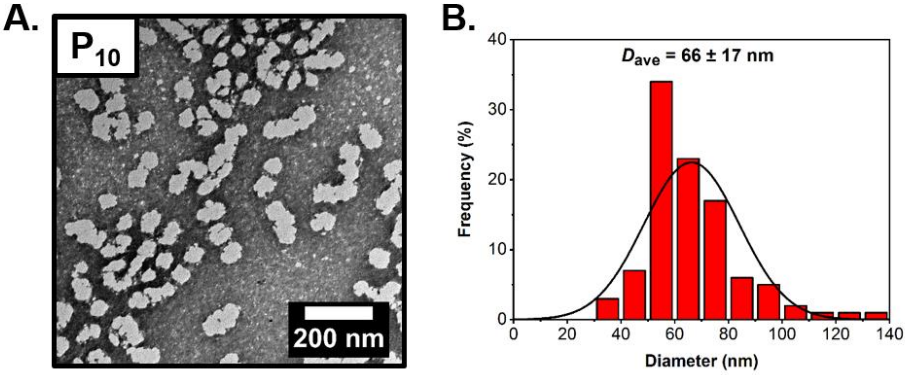

Figure S5: (A) Representative dry-state TEM image of P(DEGMA-*co*-OEGMA)-based $\mathbf{P_{10}}$ particles containing x = 10 mol% OEGMA, stained with 1 wt% uranyl acetate (UA) solution (scale bar represents 200 nm), and (B) histogram of their corresponding size distribution along with calculated average diameter, measured from particle analysis based on acquired dry-state TEM images.

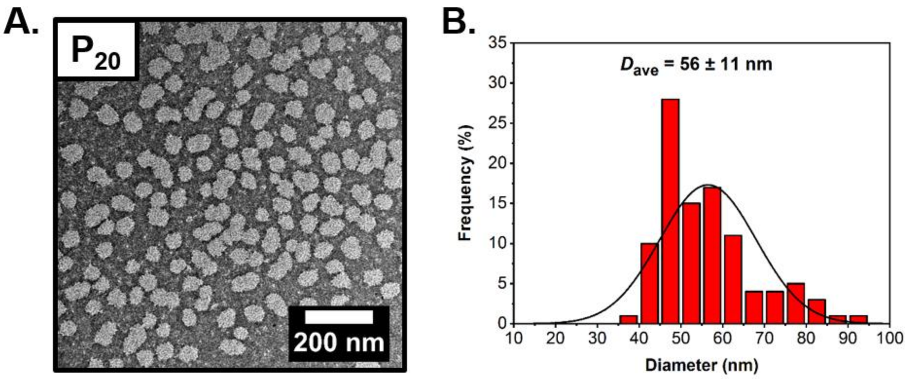

Figure S6: (A) Representative dry-state TEM image of P(DEGMA-*co*-OEGMA)-based $\mathbf{P_{20}}$ particles containing x = 20 mol% OEGMA, stained with 1 wt% uranyl acetate (UA) solution (scale bar represents 200 nm), and (B) histogram of their corresponding size distribution along with calculated average diameter, measured from particle analysis based on acquired dry-state TEM images.

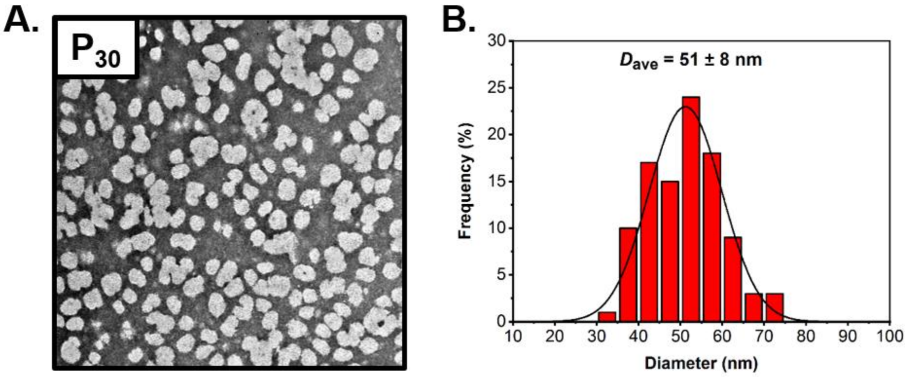

Figure S7: (A) Representative dry-state TEM image of P(DEGMA-*co*-OEGMA)-based $\mathbf{P_{30}}$ particles containing x = 30 mol% OEGMA, stained with 1 wt% uranyl acetate (UA) solution (scale bar represents 200 nm), and (B) histogram of their corresponding size distribution along with calculated average diameter, measured from particle analysis based on acquired dry-state TEM images.

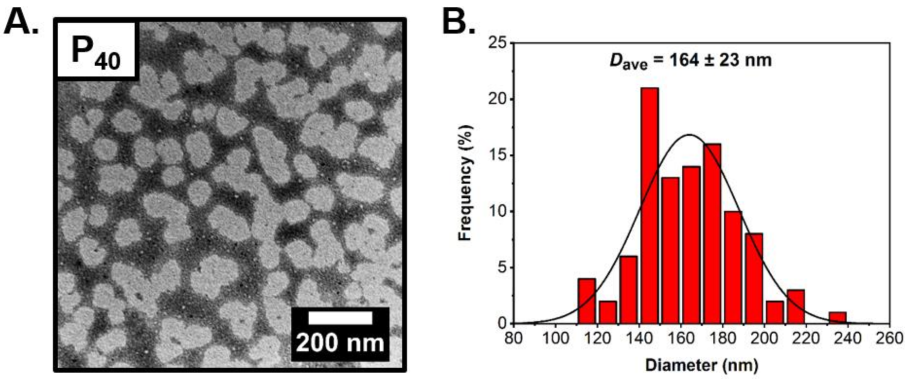

Figure S8: (A) Representative dry-state TEM image of P(DEGMA-*co*-OEGMA)-based $\mathbf{P_{40}}$ particles containing x = 40 mol% OEGMA, stained with 1 wt% uranyl acetate (UA) solution (scale bar represents 200 nm), and (B) histogram of their corresponding size distribution along with calculated average diameter, measured from particle analysis based on acquired dry-state TEM images.

Sandpack analysis of P_x particles containing x = 0, 10, 20, 30 and 40 mol% OEGMA at 5 mg mL^{-1} in 0.3 M $NaCl_{(aq)}$

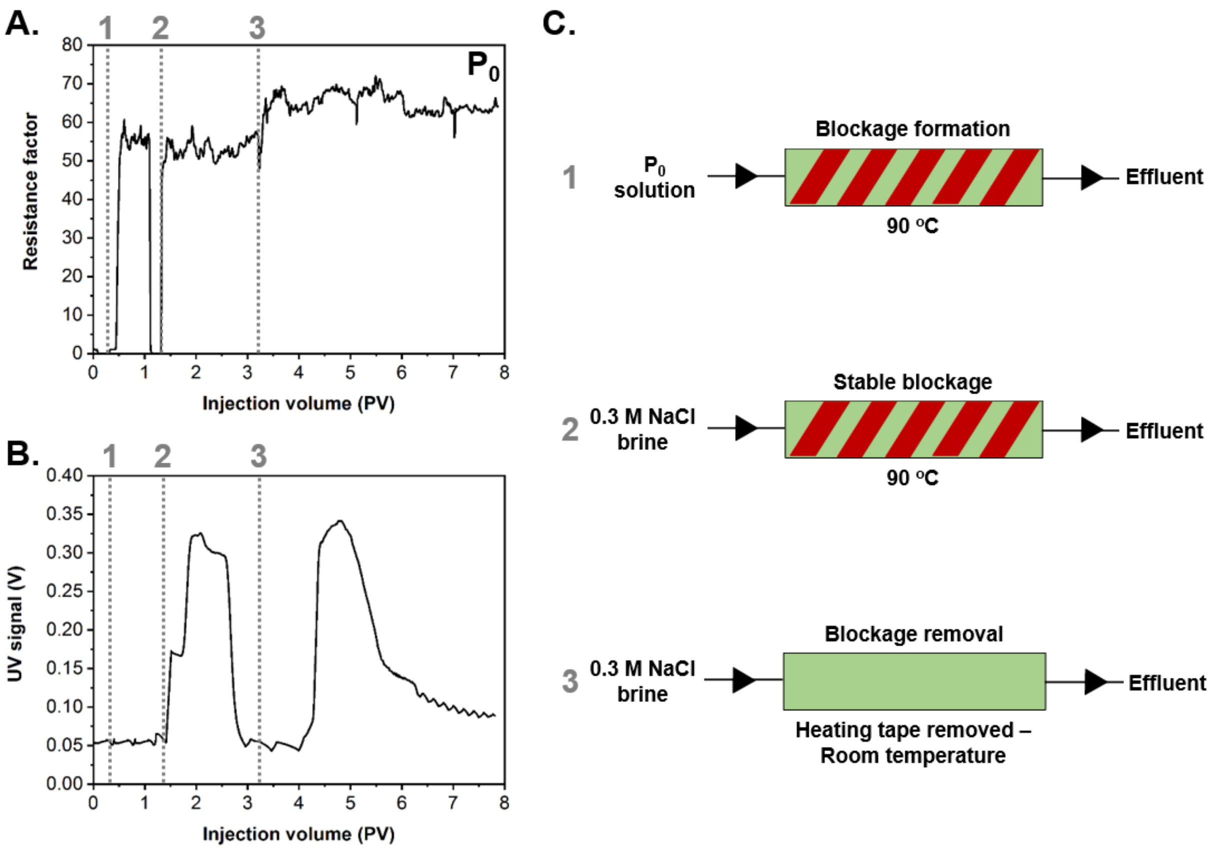

Figure S9: (A) Pressure drop profile of PDEGMA-based $\mathbf{P_0}$ particles containing x = 0 mol% OEGMA, injected at 5 mg mL^{-1} (0.3 M NaCl, pH = 5.5, flow rate = 0.1 mL min^{-1}), and (B) monitoring of UV signal of the effluent during sandpack experiments. (C) Schematic representation of the three different stages and conditions of single-column sandpack testing performed herein.

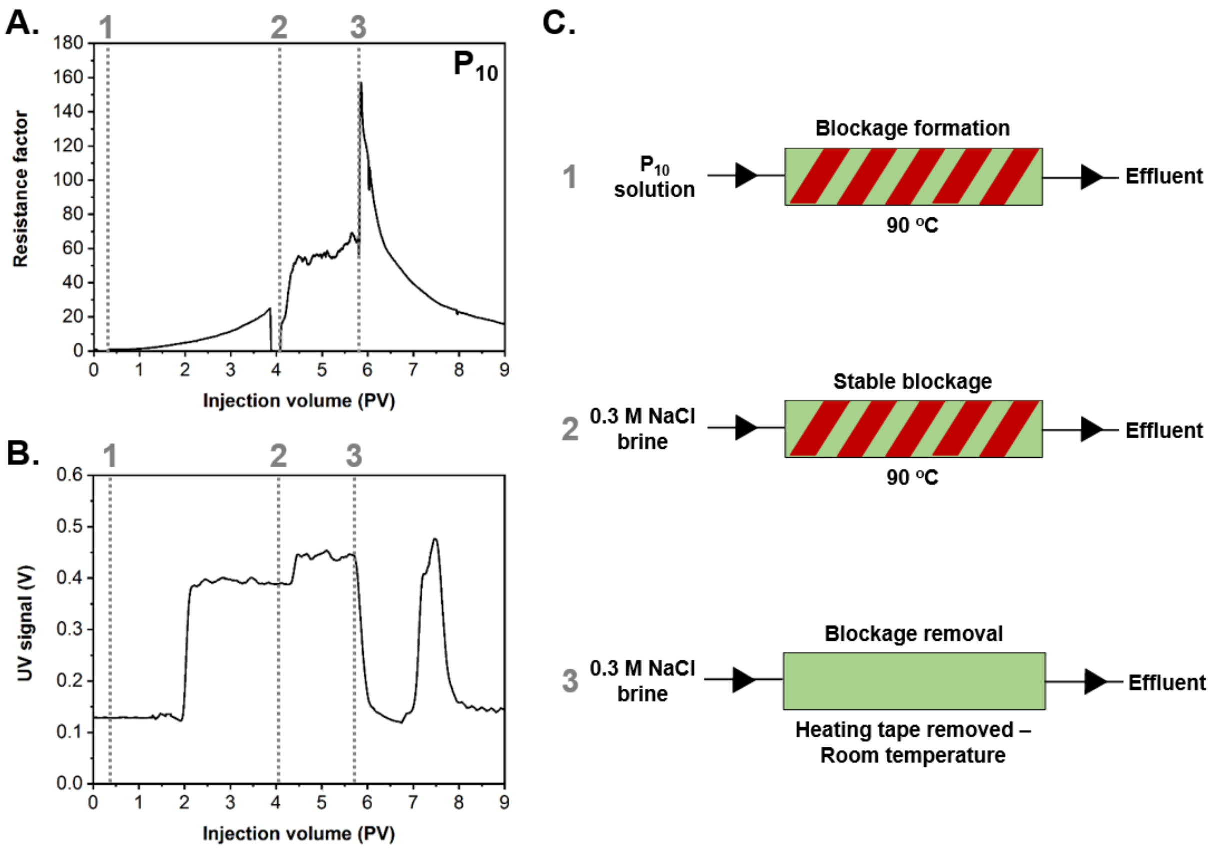

Figure S10: (A) Pressure drop profile of P(DEGMA-*co*-OEGMA)-based $\mathbf{P_{10}}$ particles containing x = 10 mol% OEGMA, injected at 5 mg mL^{-1} (0.3 M NaCl, pH = 5.5, flow rate = 0.1 mL min^{-1}), and (B) monitoring of UV signal of the effluent during sandpack experiments. (C) Schematic representation of the three different stages and conditions of single-column sandpack testing performed herein.

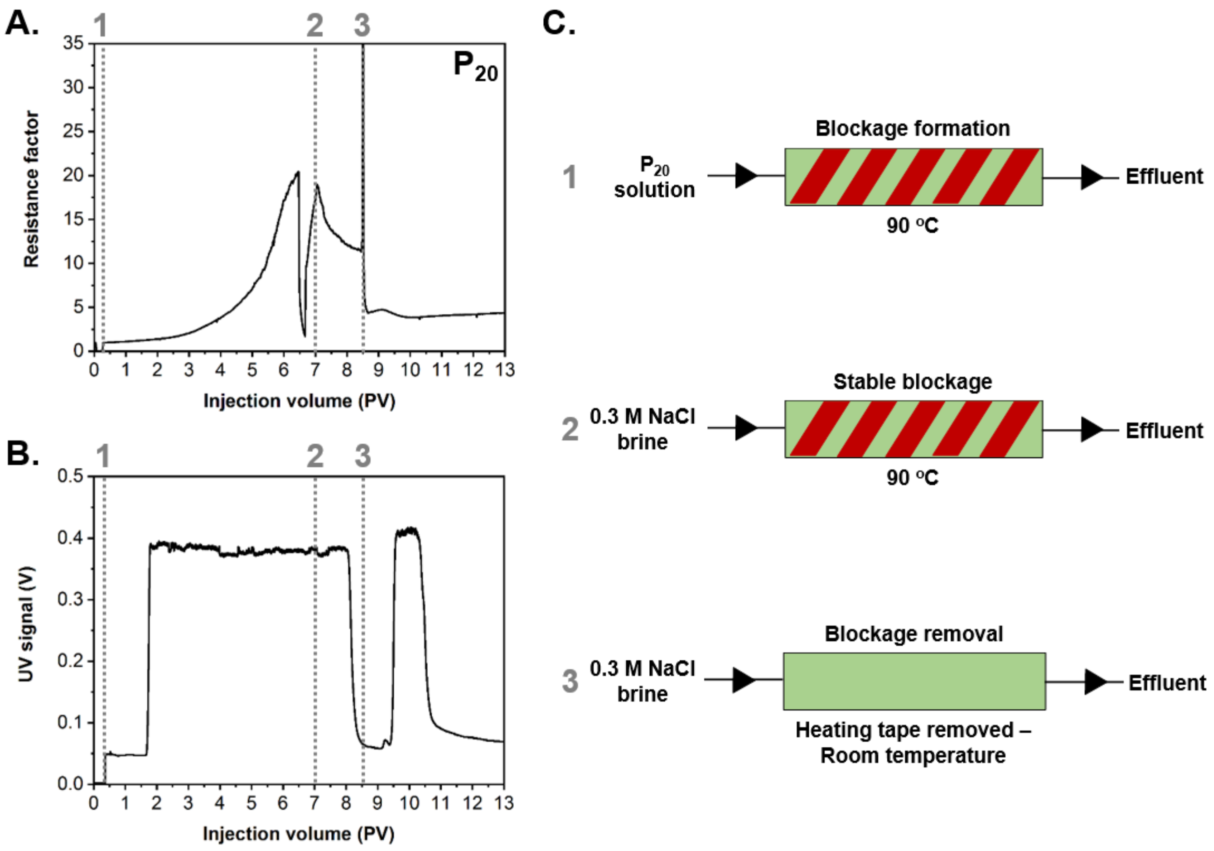

Figure S11: (A) Pressure drop profile of P(DEGMA-*co*-OEGMA)-based $\mathbf{P_{20}}$ particles containing x = 20 mol% OEGMA, injected at 5 mg mL^{-1} (0.3 M NaCl, pH = 5.5, flow rate = 0.1 mL min^{-1}), and (B) monitoring of UV signal of the effluent during sandpack experiments. (C) Schematic representation of the three different stages and conditions of single-column sandpack testing performed herein.

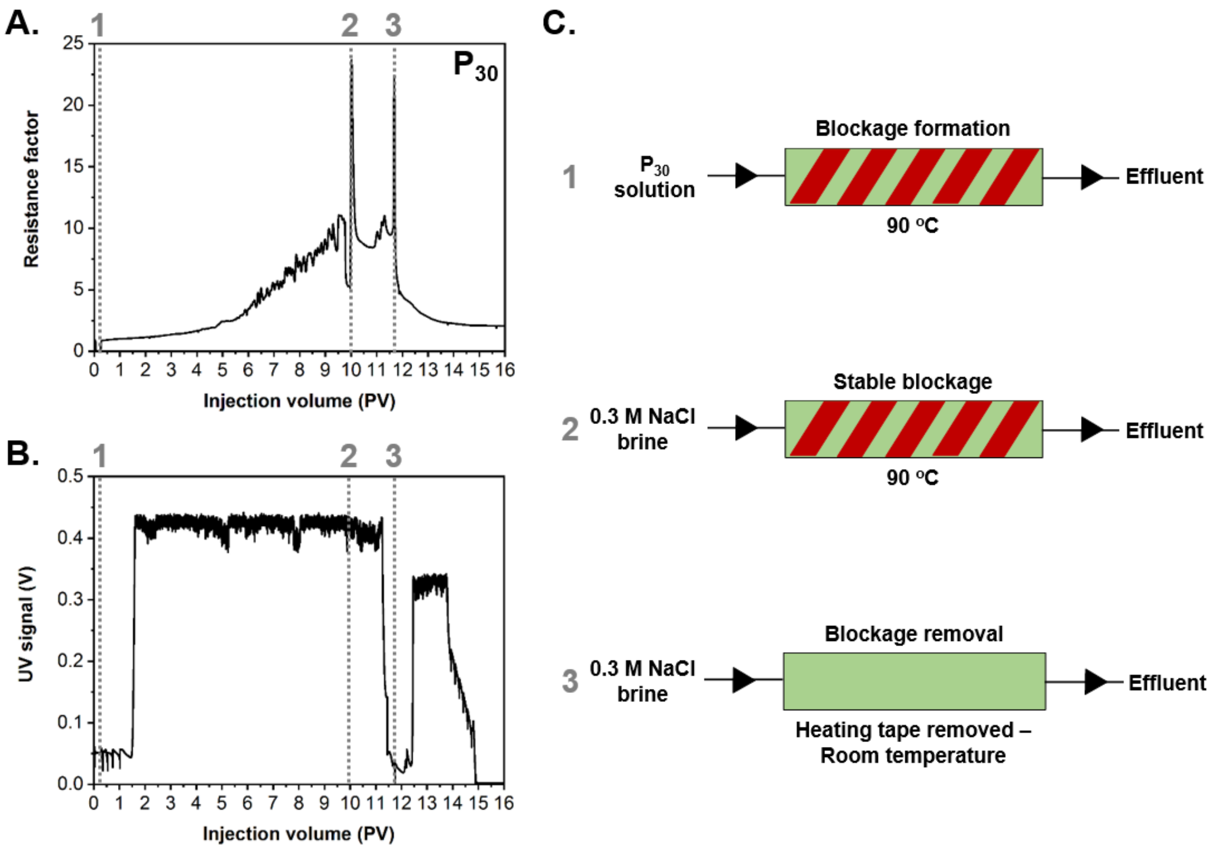

Figure S12: (A) Pressure drop profile of P(DEGMA-*co*-OEGMA)-based $\mathbf{P_{30}}$ particles containing x = 30 mol% OEGMA, injected at 5 mg mL^{-1} (0.3 M NaCl, pH = 5.5, flow rate = 0.1 mL min^{-1}), and (B) monitoring of UV signal of the effluent during sandpack experiments. (C) Schematic representation of the three different stages and conditions of single-column sandpack testing performed herein.

Variable-temperature DLS analysis of different batches of P_0 particles

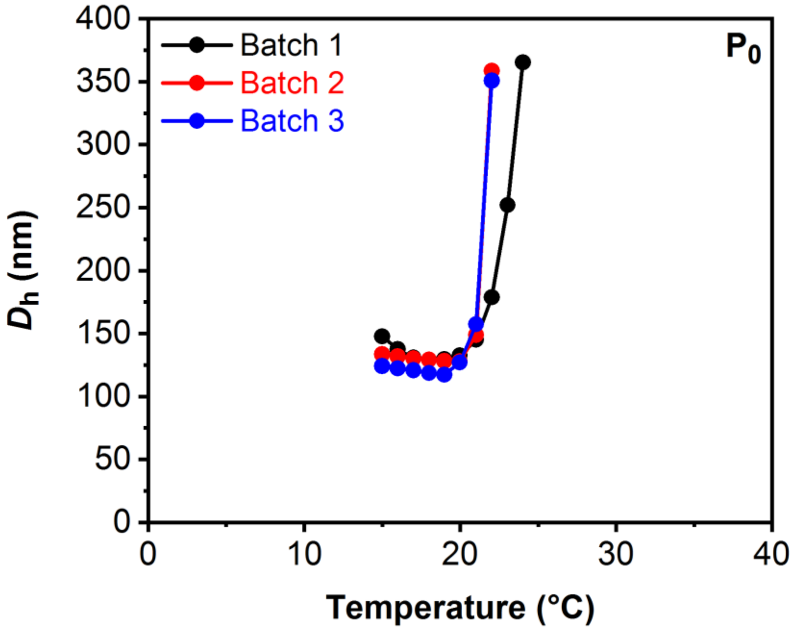

Figure S13: Evaluation of the thermoresponsive behavior of three different batches of PDEGMA-based $\mathbf{P_0}$ particles by variable-temperature DLS analysis performed from 15 to 90 °C at 1 mg mL^{-1} in 0.3 M $NaCl_{(aq)}$.
